# The composition and abundance of bacterial communities residing in the gut of *Glossina palpalis palpalis* captured in two sites of southern Cameroon

**DOI:** 10.1186/s13071-019-3402-2

**Published:** 2019-04-02

**Authors:** Jean Marc Tsagmo Ngoune, Julie Reveillaud, Guilhem Sempere, Flobert Njiokou, Trésor T. Melachio, Luc Abate, Majoline T. Tchioffo, Anne Geiger

**Affiliations:** 1 INTERTRYP, Institut de Recherche pour le Développement, University of Montpellier, Montpellier, France; 2 Faculty of Science, University of Yaoundé I, P.O. Box 812, Yaoundé, Cameroon; 3 ASTRE, INRA, CIRAD, University of Montpellier, Montpellier, France; 4 UMR Maladies Infectieuses Et Vecteurs Écologie, Génétique, Évolution Et Contrôle, IRD 224-Centre National de la Recherche Scientifique, 5290-UM1-UM2, Montpellier, France; 5 Center for Research on Filariasis and other Tropical Diseases (CRFilMT), P.O. Box 5797, Yaoundé, Cameroon; 6 Faculty of Science, University of Yaoundé I, P.O. Box 812, Yaoundé, Cameroon

**Keywords:** *Glossina*, Bacterial flora, Sleeping sickness, Nagana, Trypanosome, Metabarcoding

## Abstract

**Background:**

A number of reports have demonstrated the role of insect bacterial flora on their host’s physiology and metabolism. The tsetse host and vector of trypanosomes responsible for human sleeping sickness (human African trypanosomiasis, HAT) and nagana in animals (African animal trypanosomiasis, AAT) carry bacteria that influence its diet and immune processes. However, the mechanisms involved in these processes remain poorly documented. This underscores the need for increased research into the bacterial flora composition and structure of tsetse flies. The aim of this study was to identify the diversity and relative abundance of bacterial genera in *Glossina palpalis palpalis* flies collected in two trypanosomiasis foci in Cameroon.

**Methods:**

Samples of *G. p. palpalis* which were either negative or naturally trypanosome-positive were collected in two foci located in southern Cameroon (Campo and Bipindi). Using the V3V4 and V4 variable regions of the small subunit of the *16S* ribosomal RNA gene, we analyzed the respective bacteriome of the flies’ midguts.

**Results:**

We identified ten bacterial genera. In addition, we observed that the relative abundance of the obligate endosymbiont *Wigglesworthia* was highly prominent (around 99%), regardless of the analyzed region. The remaining genera represented approximately 1% of the bacterial flora, and were composed of *Salmonella*, *Spiroplasma*, *Sphingomonas*, *Methylobacterium*, *Acidibacter*, *Tsukamurella*, *Serratia*, *Kluyvera* and an unidentified bacterium. The genus *Sodalis* was present but with a very low abundance. Globally, no statistically significant difference was found between the bacterial compositions of flies from the two foci, and between positive and trypanosome-negative flies. However, *Salmonella* and *Serratia* were only described in trypanosome-negative flies, suggesting a potential role for these two bacteria in fly refractoriness to trypanosome infection. In addition, our study showed the V4 region of the small subunit of the *16S* ribosomal RNA gene was more efficient than the V3V4 region at describing the totality of the bacterial diversity.

**Conclusions:**

A very large diversity of bacteria was identified with the discovering of species reported to secrete anti-parasitic compounds or to modulate vector competence in other insects. For future studies, the analyses should be enlarged with larger sampling including foci from several countries.

**Electronic supplementary material:**

The online version of this article (10.1186/s13071-019-3402-2) contains supplementary material, which is available to authorized users.

## Background

In recent years, the fight against vector-borne tropical diseases has evolved towards controlling the insect vectors that transmit parasites to their human or animal hosts [1]. This control approach requires an in-depth study of the vectors in order to identify usable features involved in parasite transmission [2–4], and is increasingly performed in the context of national or international disease eradication programs. This is the case for trypanosomiasis (i.e. sleeping sickness), a neglected tropical disease caused by a protozoan parasite of the genus *Trypanosoma*, which has been targeted for elimination by the WHO and PATTEC (Pan-African Tsetse and Trypanosomiasis Eradication Campaign) [5–8]. Two subspecies, *Trypanosoma brucei gambiense* (*Tbg*) and *T. b. rhodesiense* (*Tbr*), are responsible for the chronic form of the disease in central and western Africa, and for the acute form in east Africa, respectively [9]. These pathogens are transmitted to their human host by *Glossina palpalis* and *Glossina morsitans* tsetse flies, respectively [10, 11]. Besides *Tbg* and *Tbr*, the causative agents of human trypanosomiasis, other trypanosome species including *T. b. brucei* (*Tbb*), *T. congolense* (*Tc*) and *T. vivax* (*Tv*) are transmitted to various wild or domestic animals by tsetse flies. These flies belong primarily to either the *palpalis* (west and central Africa) or *morsitans* (east Africa) groups. Among this latter group of trypanosome species, *T. congolense* (forest and savannah types) is a major cattle pathogen [12] and thus has a central role in the high economic impact of African animal trypanosomiasis (AAT) [13].

Despite the differences between *T. congolense* and *T. brucei* regarding their hosts, vectors and virulence (reviewed in [12, 14, 15]), these pathogens share a number of biological characteristics such as the need to infect two consecutive and different hosts (e.g. a tsetse fly followed by a mammal) in order to complete their life-cycle. Both trypanosomes differentiate within the fly into several forms and must undergo a maturation process to enter their metacyclic form, the only form that is infectious for the vertebrate host; both secrete proteins, some of which may be involved in their establishment within the fly or in their pathogenicity towards the vertebrate host [16–18]; both are covered with a surface protein mantel, either a variable surface glycoprotein (VSG) that covers the trypanosome bloodstream forms allowing them to evade the vertebrate host’s immune defenses [19–21], or a procyclin (procyclic acid repetitive protein) that covers the procyclic trypanosome forms hosted by the tsetse fly [22, 23]; and finally, the establishment of both species in their respective *Glossina* host is favored by the tsetse symbiont, *Sodalis glossinidius* [24, 25]. This trait of the *Sodalis* symbiont means that it is able to modulate tsetse fly vector competence, at least at the level of controlling trypanosome establishment within the fly’s gut. Therefore, this symbiont is a possible target for controlling the spread of trypanosomes, and consequently controlling the disease itself. This finding also raises an important question: if *S. glossinidius* is involved in its host vector competence, could other tsetse gut bacteria have a similar role? In this context, culture-dependent methods have been used previously to investigate the microbiome composition of tsetse flies that were sampled, regardless of their species or trypanosome infection status, in several HAT foci in Cameroon and Angola, as well as in insectary-reared flies [18, 26, 27]. Similar investigations have also been performed using either culture-dependent or non-dependent (i.e. molecular) methods on *G. fuscipes fuscipes* fly populations from Kenya [28], *G. f. fuscipes*, *G. m. morsitans* and *G. pallidipes* from Uganda [29], and *G. f. fuscipes* from Tanzania [30]. In addition, recent work has characterized the bacterial flora of *G. palpalis palpalis* flies in three foci from Cameroon (Campo, Bipindi and Fontem), demonstrating a great diversity in their bacterial flora [31]. These studies were conducted to examine the bacterial diversity of these flies, but also to identify the impact of certain bacteria (biomarkers) on their biology. However, although there are increasingly developed investigations into the bacteriome composition of tsetse flies, associating gut bacterial diversity (or the presence of specific bacteria species) with fly infection status will require further investigation. In contrast, such investigations have already been performed in the *Anopheles*-*Plasmodium* association, among others, and showed that the bacterial flora of mosquitoes, vectors of malaria, influenced the functioning of the mosquito, as well as their interaction with the *Plasmodium* during the infection [32–36].

Here, we sampled tsetse flies in two HAT foci from southern Cameroon, which were then sorted with respect to their status, trypanosome positive or negative. Thereafter, their intestinal bacteria were investigated by sequencing the V4 and V3V4 regions of the *16S* rRNA gene.

## Results

Among the 190 field flies sampled in this study, 157 were collected in Campo and 33 were collected in Bipindi. A total of 166 were trypanosome-negative (139 from Campo and 27 from Bipindi) and 24 were positive (19 from Campo and 5 from Bipindi) (Table 1).

**Table 1:** Number of *Trypanosoma congolense* (*s.l*.) simple and mixed infections in tsetse flies sampled in the different foci

Focus	Village	No. of flies analyzed	No. of flies positive with TcF (%)	No. of flies positive with TcS (%)	No. of flies positive with TcF + TcS (%)
Campo	Ipono	33	1 (3.03)	0 (0)	1 (3.03)
	Beach	74	6 (8.10)	0 (0)	4 (5.40)
	Mabiogo	51	1 (1.96)	1 (1.96)	5 (9.80)
	Total Campo	158	8 (5.06)	1 (0.63)	10 (6.32)
Bipindi	Bidjouka	17	1 (5.88)	0 (0)	0 (0)
	Lambi	10	4 (40)	0 (0)	0 (0)
	Ebimimbang	5	0 (0)	0 (0)	0 (0)
	Total Bipindi	32	5 (15.62)	0 (0)	0 (0)
Total		190	13 (6.84)	1 (0.52)	10 (5.26)
Global		190	24 (12.63)		

*Abbreviations*: TcF, *T. congolense* “forest” type; TcS, *T. congolense* “savannah” type; mixed infection, flies simultaneously positive for both parasites (TcF + TcS)

A total of 6,233,517 paired-end reads were generated, including 4,234,788 and 1,776,855 paired-end reads from the sequencing of the V4 and V3V4 regions, respectively. From this, 4,185,626 reads and 1,696,768 reads were successfully aligned for the V4 and V3V4 regions, respectively, representing a total of 5,882,394 reads (94.36%), with an average sequencing depth of 22,263 (± 2372) paired-end reads per sample. Ninety-seven percent of these sequences passed the filter barrier described above, which indicated the good quality of the sequences and thus the efficiency of the sequencing. Ten taxa were taxonomically assigned at the genus level, nine of which were made possible by region V4, and seven by region V3V4 (Table 2, Additional file 1: Table S1).

**Table 2:** Summary of Illumina tags among the V4 and V3V4 *16S* rRNA regions

	Global	V4	V3V4
Raw, *n*	6,233,517	4,435,468	1,776,855
Merged, *n* (%)	5,882,394 (94.36)	4,185,626 (94.36)	1,696,768 (94.36)
Dereplicated, *n* (%)	5,771,010 (98.10)	4,107,883 (98.14)	1,663,127 (99.18)
Filtered, *n* (%)	5,641,372 (97.75)	4,082,771 (95.32)	1,558,601 (97.74)
Mean ± SD	22,263 ± 2372	22,056 ± 2372	8354 ± 2372
OTUs (genus level)	10	9	7

*Abbreviation*: SD, standard deviation; OTUs, operational taxonomic units

A rarefaction analysis that was performed to verify the sequencing depth (and thus ensure the description of the quasi-totality of the OTUs present in the samples) showed that the curves reached saturation using the V3V4 region, demonstrating that the sequencing effort was sufficient to characterize all OTUs (Additional file 2: Figure S1a). However, rarefaction curves resulting from the sequencing of the V4 region (Additional file 2: Figure S1b) did not show a similar saturation suggesting future studies might require deeper sequencing with an Illumina HiSeq system.

### Global characterization of bacterial populations in field *Glossina palpalis palpalis*

**Fig. 1:**
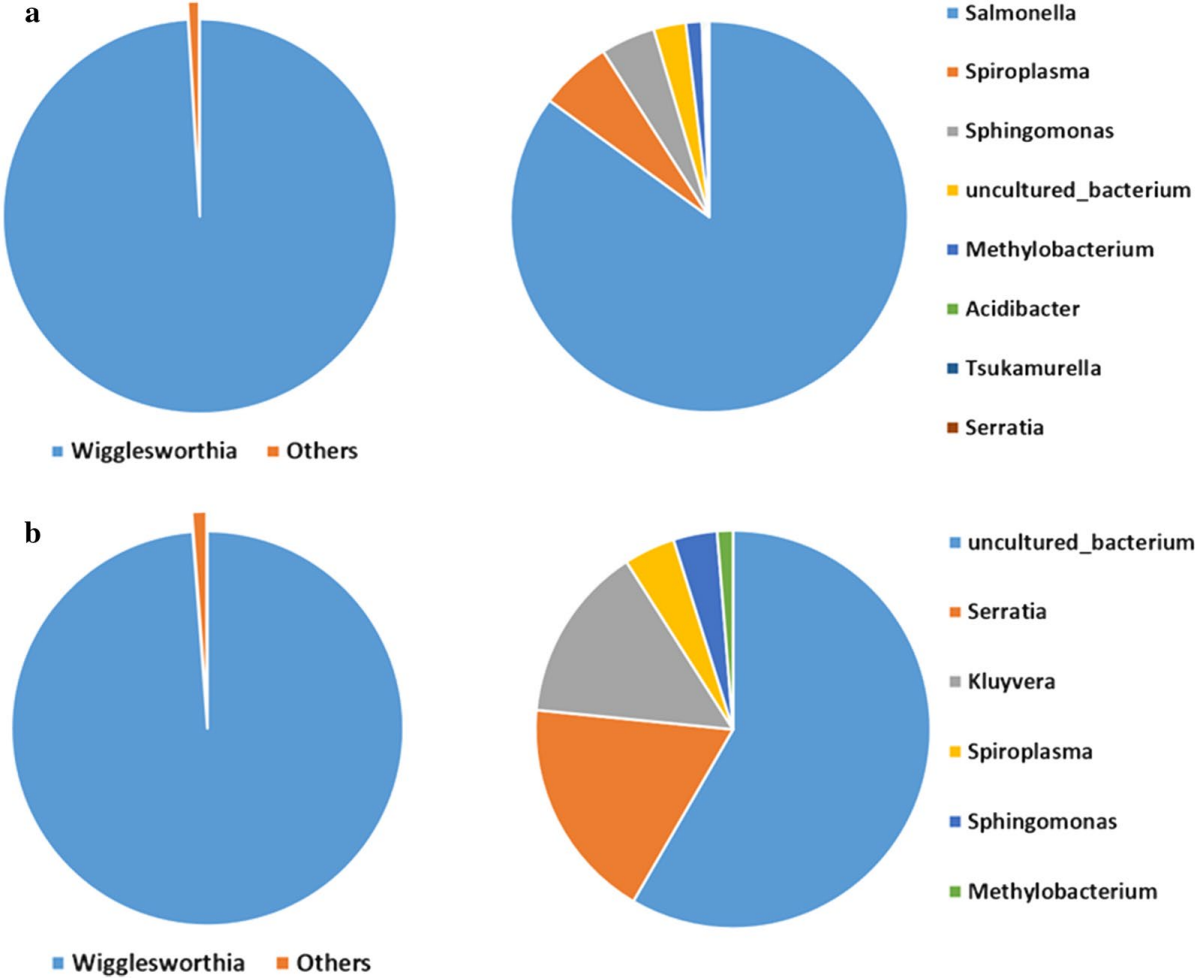
Quantitative representation of the whole bacterial community (left) and without *Wigglesworthia* (right). Results are presented for the sequencing of the V4 (**a**) and V3V4 (**b**) regions of the *16S* ribosomal RNA gene

Based on the V4 region sequencing results, the bacterial population found in flies collected in the Campo and Bipindi foci (regardless of their infection status) was distributed among eight identified and one unidentified taxa. The genus *Wigglesworthia* was the major representative, with 99.05% relative abundance in the total microbiome (Fig. 1a, Table 3). The over-representation of this genus in the bacterial flora was confirmed when sequencing the V3V4 region, where its abundance reached 98.79% (Fig. 1b, Table 3). This indicates that the other bacterial genera were present in much lower abundance, i.e. a shared maximum abundance of about 1% (0.949% or 1.205%, depending on the *16S* rRNA sequenced region) (Fig. 1a, b; Table 3). Some of the identified genera displayed similar abundances, regardless of the sequenced region; this was the case for *Spiroplasma* (0.056 and 0.050% based on V4 and V3V4 sequencing, respectively), *Sphingomonas* (0.042 *vs* 0.043%) and *Methylobacterium* (0.012 *vs* 0.015%). In contrast, two bacteria displayed a higher abundance when analyzed by the V3V4 region than with the V4 region: *Serratia* (0.218 *vs* 0.001%) and an unidentified bacterium (0.704 *vs* 0.024%). Finally, three bacterial genera, *Salmonella* (abundance: 0.8%), *Acidibacter* (abundance: 0.0022%) and *Tsukamurella* (abundance: 0.0021%) could only be identified by sequencing the V4 region, whereas the genus *Kluyvera* (abundance: 0.173%) was identified only by sequencing the V3V4 region (Table 3).

**Table 3:** Global distribution of bacteria identified by sequenced region

Genus	V4 region	V3V4 region
*Wigglesworthia*	99.0501645	98.7931867
*Salmonella*	0.80869642	0
*Spiroplasma*	0.05616735	0.05055764
*Sphingomonas*	0.04217868	0.04317422
Uncultured bacterium	0.02485825	0.704
*Methylobacterium*	0.01231098	0.01551769
*Acidibacter*	0.00222117	0
*Tsukamurella*	0.00215029	0
*Serratia*	0.00125236	0.21862423
*Kluyvera*	0	0.17326002

### Variation in bacterial flora of the flies according to foci

**Fig. 2:**
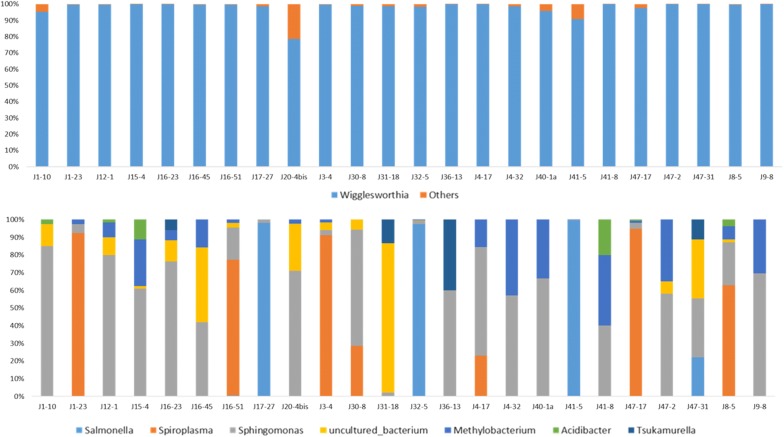
Relative bacterial abundance according to tsetse origin. Results are presented for the Campo focus. The top and bottom parts of the figure represent the relative abundance of bacteria with or without *Wigglesworthia*, respectively. The 24 *Glossina* flies presented in this figure were randomly chosen as representatives of the 139 flies sampled in the Campo focus. Others: all other bacteria besides *Wigglesworthia*

In order to determine whether there was any variation in the bacterial flora of tsetse flies according to their origin (i.e. Campo or Bipindi), we systematically analysed the number of taxa and their abundance present in each fly across all flies sampled in Campo, and compared this with similar data recorded for the flies sampled in Bipindi. In order to see the impact of a particular condition (infection status or site effect) on bacterial flora composition, we used the results from the V4 region, since this region allowed identifying more taxa than V3V4 (except for the genus *Kluyvera*, which was only present in trace amounts and can thus be neglected). Our analysis showed that the genus *Wigglesworthia* was highly dominant (99.06%) in Campo, as shown in Fig. 2, Additional file 3: Figure S2 and Table 4. After *Wigglesworthia*, the other genera represented less than 1% of the mean abundance of the bacteria flora: *Sphingomonas* (0.43%); *Methylobacterium* (0.185%); an unidentified bacterium (0.166%); *Salmonella* (0.077%); *Spiroplasma* (0.067%); *Acidibacter* (0.007%); and *Tsukamurella* (representing 0.001% of the bacterial flora of flies sampled in Campo). The genus *Tsukamurella* was only identified in the Campo focus.

**Table 4:** Summary of bacterial genera abundance according to the different conditions (infection status and origin of the tsetse flies). Values marked in bold denote bacteria for which the description was not possible due to low abundance (< 0.001)

Genus	Uninfected (%)	Infected (%)	Bipindi (%)	Campo (%)
*Wigglesworthia*	98.5079006	99.3791479	96.413207	99.0650593
*Salmonella*	0.6596359	**0.00080507**	3.04102797	0.07725353
*Spiroplasma*	0.05101862	0.17293821	0.06288522	0.06713508
*Sphingomonas*	0.44185363	0.16720427	0.29148198	0.43059226
Uncultured bacterium	0.13092041	0.19633577	0.00536955	0.16628591
*Methylobacterium*	0.1839627	0.0748723	0.09546668	0.18531633
*Acidibacter*	0.02332266	0.0010195	0.08675359	0.00708811
*Tsukamurella*	**0.000098538**	0.00767699	**0**	0.00126966
*Serratia*	0.00128691	**0**	0.00380803	**0**

**Fig. 3:**
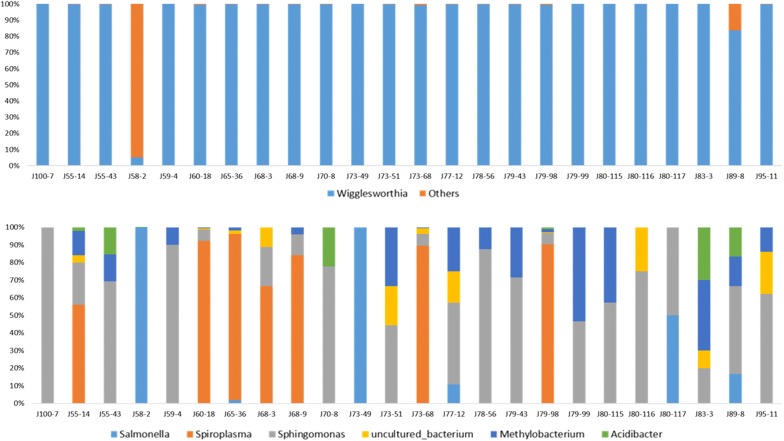
Relative bacterial abundance according to tsetse origin. Results are presented for the Bipindi focus. The top and bottom parts of the figure represent the relative abundance of bacteria with or without *Wigglesworthia*, respectively. The 24 *Glossina* flies presented in this figure were randomly chosen as representatives of the 27 flies sampled in the Bipindi focus. Others: all other bacteria besides *Wigglesworthia*

**Fig. 4:**
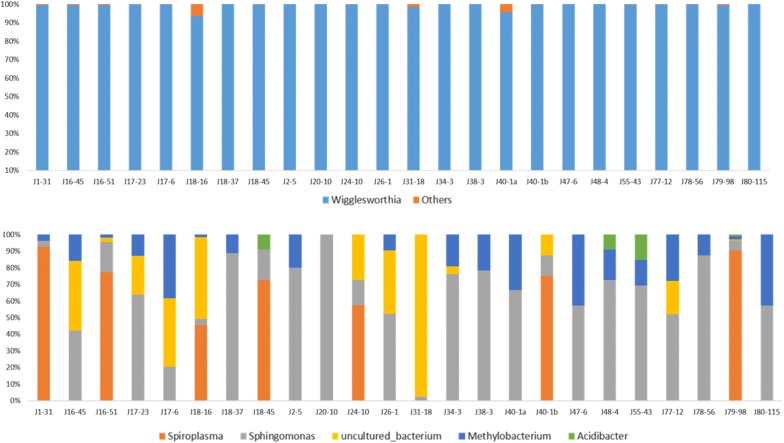
Relative abundance of bacteria in trypanosome-positive flies. The top and bottom parts of the figure represent the relative abundance of bacteria with or without *Wigglesworthia*, respectively. All trypanosome-positive flies were taken into account in this figure. Others: all other bacteria besides *Wigglesworthia*

**Fig. 5:**
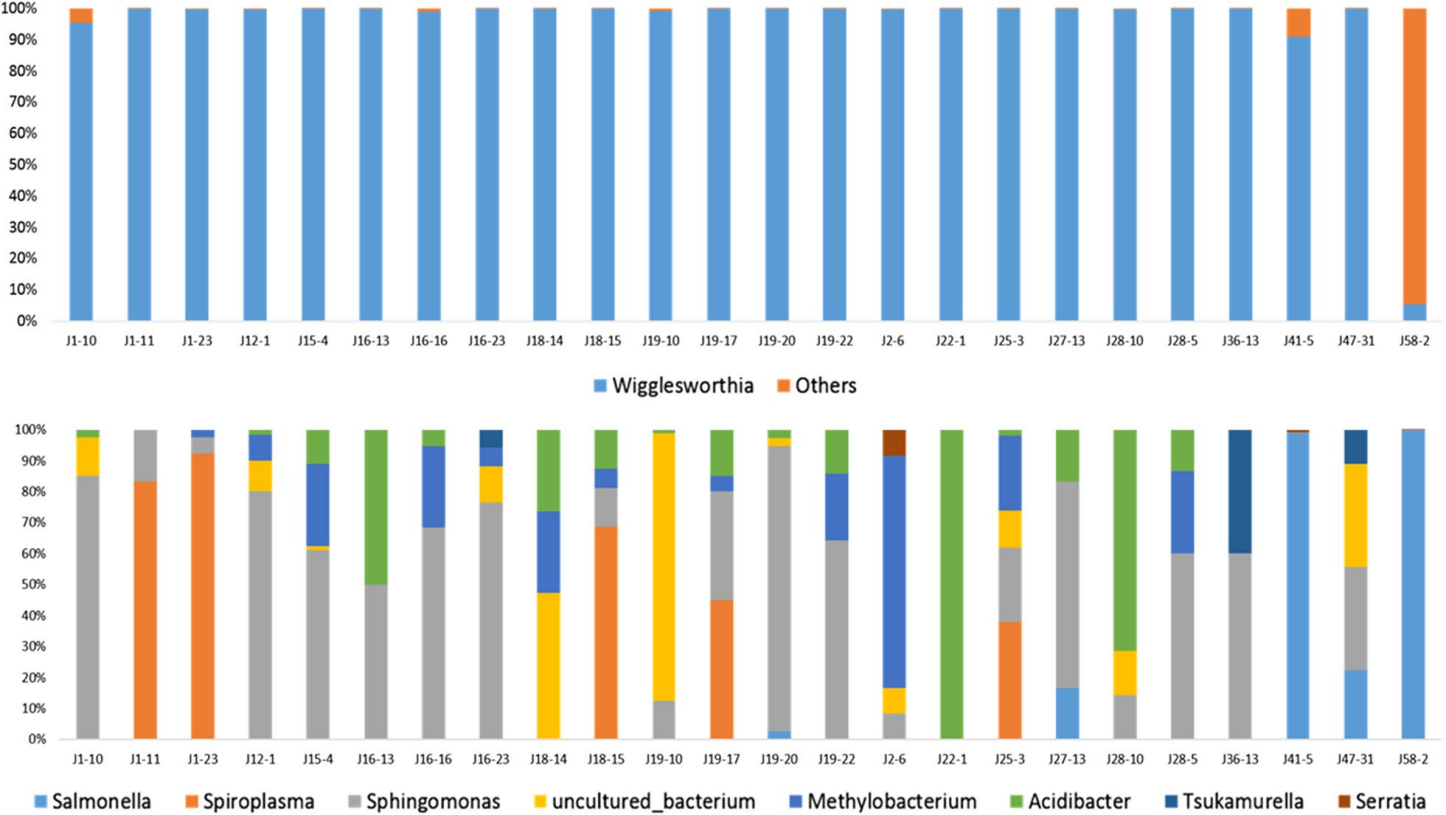
Relative abundance of bacteria in trypanosome-negative flies. The top and bottom parts of the figure represent the relative abundance of bacteria with or without *Wigglesworthia*, respectively. The 24 uninfected *Glossina* flies presented in this figure were randomly chosen as representatives of the total 166 sampled trypanosome-negative flies. Others: all other bacteria besides *Wigglesworthia*

Similarly, in the Bipindi focus (Fig. 3, Additional file 3: Figure S2, Table 4), *Wigglesworthia* displayed a prominent abundance (96.41%) in contrast to *Salmonella* (3.04%), *Sphingomonas* (0.291%), *Methylobacterium* (0.094%), *Acidibacter* (0.086%), *Spiroplasma* (0.062%) and *Serratia* (0.0038%) (Fig. 4, Fig. 5; Additional file 4: Figure S3; Table 4).

### Association between *Trypanosoma* and tsetse bacterial composition

Regarding trypanosome-positive tsetse flies, the bacterial population identified using the V4 assay was composed of *Wigglesworthia* (99.37%), an unidentified bacterium (0.19%), *Spiroplasma* (0.17%), *Sphingomonas* (0.16%), *Methylobacterium* (0.07%) and *Acidibacter* (0.001%) (Fig. 4, Additional file 3: Figure S2, Table 4). The same genera were recovered when exclusively investigating trypanosome-positive flies from the Campo focus microbiome, as well as trypanosome-positive flies from the Bipindi focus microbiome (Fig. 4, Additional file 3: Figure S2, Table 4).

The genus *Sodalis* was found in trace amounts in the sequencing results, although the criteria for bacterial enrollment used in this study did not allow for its description in the flora due to its extremely low prevalence and abundance.

### Bacterial diversity in flies (alpha diversity)

**Fig. 6:**
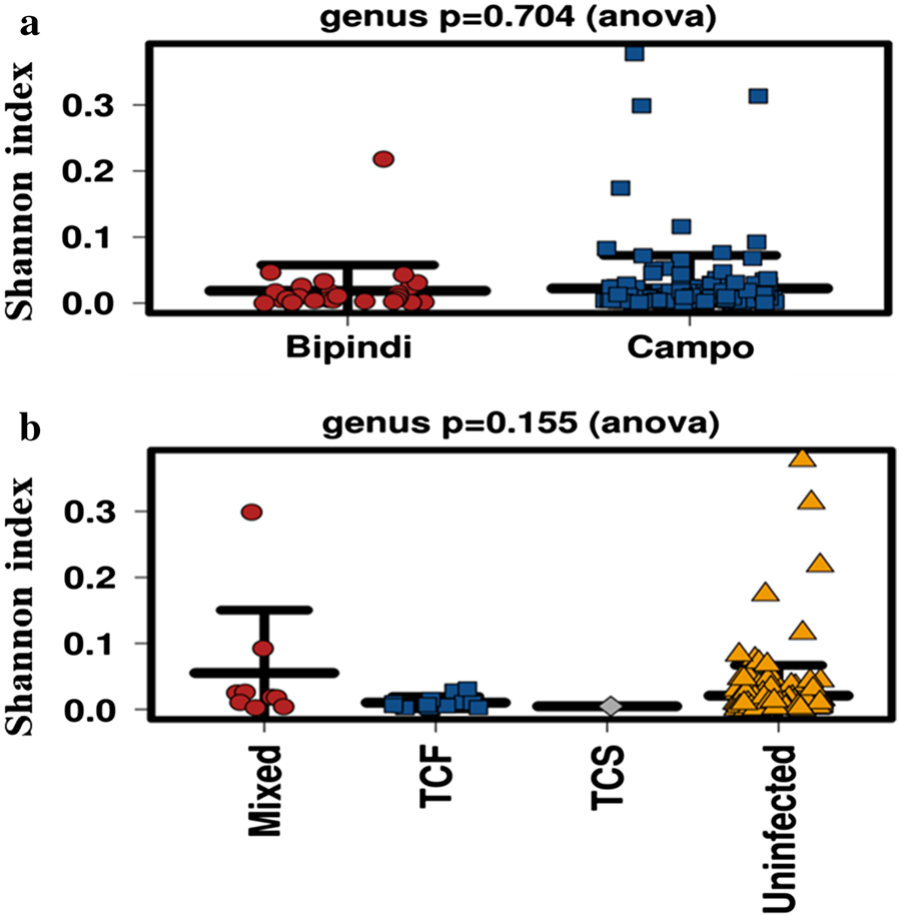
Comparison of bacterial diversity. Comparisons are presented according to tsetse origin (Campo *vs* Bipindi focus) (**a**) and fly status (trypanosome-positive *vs* negative) (**b**). *Abbreviations*: TCF, *Trypanosoma congolense* forest; TCS, *Trypanosoma congolense* savannah

No significant difference was observed between the diversity (richness and evenness) of the bacteria identified in flies from Campo and those from Bipindi (Shannon index, *P* = 0.704) (Fig. 6a). Likewise, no significant difference was observed for bacterial flora diversity between trypanosome-positive and negative flies (Shannon index, *P* = 0.155) (Fig. 6b), suggesting a similar estimated diversity for Campo and Bipindi flies as well as for trypanosome-positive and negative flies. All comparisons were performed with a threshold of α = 0.05.

### Multivariate analysis (beta diversity)

In the non-normalized abundance table (providing the number of reads per sample) (Additional file 5: Table S2), even though *Wigglesworthia* predominates, we still count between 2041 and 0 reads per sample for non-*Wigglesworthia* bacteria. Thus, a principal components analysis (PCA) using the Bray-Curtis index was performed, taking into account the bacterial composition as well as their abundance in the different samples. Data indicated that there were no significant differences between the flies in terms of the composition and structure of their bacterial flora, regardless of their infection status (Additional file 4: Figure S3b) or sampling site (Additional file 4: Figure S3a). The lack of significant difference shown by the PCA was confirmed by a permutational multivariate analysis of variance (PERMANOVA), for which non-significant *P*-values of 0.73 and 0.17 were obtained for the home and infection status parameters, respectively. In addition, PCA using the Jaccard diversity index (instead of the Bray-Curtis index) was also performed (Additional file 6: Figure S4) in order to take into account the presence/absence of bacteria instead of the relative abundance. Both graphs generated similar results.

No biomarkers were found for the different conditions studied (foci and infection status), using Lefse, confirming the fact that there was no significant difference between flies from both foci and between trypanosome-positive and negative flies.

However, a simple differential description of bacteria between conditions (presence/absence) allowed identification of the genera *Salmonella* and *Serratia* only in uninfected flies, suggesting these could represent potential biomarkers of this condition and require further investigation (Fig. 5, Additional file 3: Figure S2, Table 4).

## Discussion

Attempting to identify biological factors and mechanisms controlling fly infection in HAT or AAT foci needs to move from the laboratory to the field, i.e. from artificially trypanosome-infected insectary reared tsetse flies to field-collected and naturally infected flies. This raises difficulties resulting from moving from a controlled system to an uncontrolled. Regarding, for example, the status (trypanosome-infected or uninfected) of field-collected flies, we consider as being infected any tsetse fly whose total DNA extracted from the intestine (a mix of DNA from the fly and from microorganisms, parasites, etc., that it may harbor) positively responds to PCR performed with trypanosome-specific primers. However, this does not provide the infection background. Did it occur recently or not? Did it result from the ingestion of several meals of trypanosome-contaminated blood? Did it result from a contaminated meal following an uncontaminated one? Each sampled fly has probably had a particular life course which, at the final analytical step, may have a “smoothing” effect, especially on the statistical significance of the results. Such investigations must nevertheless be undertaken in order to detect at least trends that will allow future progress.

To our knowledge, the present study is the first to use both the V3V4 and V4 regions of the small subunit of the *16S* ribosomal RNA gene to characterize the intestinal bacterial flora of tsetse flies sampled in the trypanosomiasis foci of southern Cameroon (Campo and Bipindi), using flies naturally positive (or not) with *Trypanosoma congolense*. Importantly, we provide new insight into how the bacterial flora composition depends on fly infection status or sampling site. Our findings reveal that the bacterial population harbored by *G. p. palpalis* is dominated by the genus *Wigglesworthia* (greater than 99% relative abundance), which is not surprising since it is the obligate mutualist symbiont of tsetse flies [29, 37, 38]. Furthermore, our results are in complete agreement with two recent reports that used *16S* rRNA deep sequencing [27, 29] and demonstrate that the abundance of *Wigglesworthia* is greater than 99% in flies harvested from villages in Uganda, and close to 94% in flies sampled in Campo, respectively. *Wigglesworthia* is a member of the phylum Proteobacteria, which represents the vast majority of bacteria found in association with insects; these taxa allow insects to manage their energy [39]. These bacteria have been described mainly in *Anopheles*, the major vector of malaria [33, 35]. This tropical disease, like trypanosomiasis, is caused by protozoa and is transmitted by hematophagous insects [40]. In addition to Proteobacteria, these two vectors can share highly similar bacterial flora.

The higher number of bacterial taxa at Campo than Bipindi could be due to the fact that more samples from Campo were used in this study (*n* = 157) than from Bipindi (*n* = 33). Aksoy et al. [29] previously described a similar result in tsetse flies from Uganda, in which the region of Kaberamaido yielded the largest number of samples and displayed the highest number of bacterial taxa.

Globally, no significant differences were found between bacterial populations in the flies, depending on the foci. This could be due in part to the fact that the villages of Campo and Bipindi, both historical foci of sleeping sickness, are located in Cameroon’s South Region [41]. Therefore, the two foci may share the same eco-climatic features with a Guinean-like climate characteristic of the Congo Basin’s forests. These results are also in agreement with Jacob et al. [31], who demonstrated that the bacterial composition of flies collected at the Campo and Bipindi foci were not significantly different. In addition, this result is similar to that of Tchioffo et al. [36] and Gimonneau et al. [33] who did not show any significant differences between the bacterial flora of the mosquitoes *Anopheles coluzzi* and *Anopheles gambiae* in Cameroon. Until recently, these sister species were described as the same species, i.e. *A. gambiae* (*s.l.*) [32, 42, 43], suggesting that they share the same morphological and biological characteristics. In line with these studies, Aksoy et al. [27] revealed differences in microbial composition between genetically distinct tsetse fly populations. This could be due to the fact that microbial communities are associated with tsetse species (*G. fuscipes fuscipes*, *G. morsitans morsitans* and *G. pallidipes*) that are commonly found in different biotic and abiotic habitats, and which originated from regions separated by great distance in Uganda. However, although Campo and Bipindi are comparable in that they share the same environmental characteristics (climate, flora, human activities), they do display some peculiarities regarding fauna that have been shown to act as potential reservoirs for diverse trypanosome species [44, 45]. In their reports, Farikou et al. [44] and Njiokou et al. [45] did not make a comparative inventory of the fauna present in the two foci, they only compared the origins of the blood meals taken by the tsetse flies that were trapped there.

Nevertheless, the investigations provide some information on the diversity of the fauna present in both foci. In addition to humans, several domestic or wild mammals and some reptilians were identified: pig (domestic and wild), coat, sheep (two species), duiker (three species), antelope, monkey, snake (python) and turtle. Most of these species are present in both foci. However, based on the frequencies of the meals taken by the tsetse flies on the different species, Bipindi and Campo differ from each other in their respective densities of the population of given species. For example, in Bipindi most of the blood meals were from pigs (66.7 *vs* 23.5% from humans), while in Campo blood meals from humans were predominant (62.9 *vs* 22.7% from pigs). This indicates the existence of significant differences in the respective human and pig population densities in Bipindi as compared to Campo. Similar differences were recorded regarding the antelope where, in 2004, 18% of the blood meals were from the antelope in Campo, compared to only 1.5% in Bipindi [46]. Finally, comparing the feeding patterns recorded in 2008 [44] to those recorded in 2004 [46] showed significant differences that may indicate that the population densities of the different species can evolve rapidly with time in a given ecosystem. It has been reported that tsetse flies may not be strictly hematophagous; they can feed on a broad range of nectar plants [47] and thus acquire nectar bacteria. They may also become contaminated through contact with bacteria present on the skin of humans and animals when ingesting a blood meal [48]. This could explain why the tsetse fly can be contaminated by a large diversity of bacteria and why the bacteriome composition could vary according to differences in fauna availability for their blood meal. It could also partly explain why the genus *Tsukamurella* was only identified in Campo in our study.

Global statistical analyses did not show any significant association between the bacterial flora of flies and *T. congolense* infection. The lack of bacterial biomarkers in this study precluded us from opening new avenues of research about their possible impact on the biology of the flies, thus improving biological strategies to fight against these vectors. However, the absence of biomarkers could be explained by the overwhelming presence of the genus *Wigglesworthia*, which would not only prevent the identification of biomarkers, but also the effective amplification of low abundance or rare bacterial genera. The genus *Sodalis*, for example, could not possibly be described because of its very low abundance (below the threshold retained for the study). It is possible other bacteria well known in the literature, as well as new bacteria that could represent potential biomarkers, are hidden by such dominant genera.

However, a simple differential description of bacteria between *Trypanosoma*-positive and -negative flies showed that *Salmonella* and *Serratia* were detected in trypanosome-negative flies only, suggesting a possible association between these bacteria and the lack of fly infection with *Trypanosoma*, or conversely, that the presence of the parasite could influence the bacterial flora composition of the flies.

*Salmonella* was the predominant genus among bacteria specific to Tc-negative flies (0.659%), suggesting a possible role in protecting flies against trypanosome presence. However, no parallel could be found with other infected insects.

*Serratia marcescens* has been previously shown to secrete trypanolytic compounds and to reduce the establishment of *T. cruzi* in the midgut of its vector *Rhodnius prolixus* [49]. Bando et al. [50] recently isolated *S. marcescens* from wild insect populations in Burkina Faso, whose features (variation and structure of bacteria cells) directly correlate with its ability to inhibit *Plasmodium* development in *Anopheles* sp. Another *Serratia* species, *Serratia odorifera*, has been shown to enhance the susceptibility of *Aedes aegypti* to chikungunya virus [51], as well as its susceptibility to dengue-2 virus [52]. In addition, *Salmonella* sp. [53] and *Serratia* sp. [54] have been shown to induce dysbiosis and inflammation has been observed in both cases; however, the effect on mammals and that on insects might be due to different species or pathotypes. These different results show the complexity of the interactions between *Serratia* strains and vector hosts, and reinforce the need to better understand the association of *Serratia* with tsetse flies. They illustrate the need to confirm and characterize the *Salmonella* and *Serratia* species and/or the genetic diversity of species strains isolated from our fly samples.

Our results indicate, as demonstrated by Boissière et al. [32], that sequencing the V4 region is more effective than the V3V4 region in characterizing midgut bacterial diversity, since it allows the identification of most bacterial genera.

However, sequencing the V3V4 region did allow us to confirm the results obtained through V4 sequencing, and to also identify one other species (*Kluyvera*) that was not found when sequencing the V4 region. In line with these observations, Kozich et al. [55] reported that out of the three *16S* ribosomal RNA regions (V3V4, V4 and V4V5), the V4 region generated the lowest sequencing error (0.01%), making it the most appropriate region for identifying OTUs.

Although *Sodalis* is a secondary endosymbiont of tsetse flies, its relative abundance was too low to properly describe it here. This could be due to the fact that it has a more general tropism beyond the intestine and thus can be localized in several other tissues, both extracellularly and intracellularly, including the salivary glands and the hemocoel [38]. However, *Sodalis* has never been observed to be very abundant in tsetse fly midguts, with maximum reported abundances of around 0.26% [29] and 0.06% [31].

## Conclusions

In conclusion, this study provides new evidence that *Wigglesworthia*, the obligatory primary symbiont of tsetse flies, is the predominant genus within the tsetse fly intestinal flora. No significant differences were found between the bacterial composition of tsetse flies positive in trypanosome and negative, nor in function of their sampling sites (Campo or Bipindi). A deeper sequencing of bacteria communities associated with the flies will allow for further investigation of the diversity of the non-symbiotic flora and improve the significance of results. A differential investigation demonstrated that the genera *Salmonella* and *Serratia* were only described in uninfected flies, suggesting a possible association of these taxa to the refractory status of flies. Finally, the V4 region of the small subunit of the *16S* ribosomal RNA gene proved to be the most effective region for our metabarcoding analysis. We suggest future research should aim at unraveling the interactions between the less abundant and rare flies bacterial taxa and trypanosomes. In addition, deep sequencing should be performed on teneral flies in order to identify bacteria already present in their gut. Owing to the fact that tsetse flies are viviparous, exogenous bacteria cannot have contaminated the teneral flies’ gut. This approach may allow the identification of bacteria inherited from their mother fly.

## Methods

### Sampling areas

Tsetse flies were sampled in May and June 2015 in two active HAT foci (Campo and Bipindi; the two foci are about 150 km apart from each other), located in the south region of Cameroon. The Campo focus (2°20′N, 9°52′E) is located on the Atlantic coast and extends along the Ntem river. The HAT National Control program that surveys Campo once a year diagnosed 61 novel HAT cases between 2001 and 2011. HAT was still active one year after completing the sampling campaign, since 2 novel cases were passively identified in 2016 [56]. The Bipindi focus (3°2′N, 10°22′E), has been known since 1920 [57]; it has a typical forest bioecological environment, including equatorial forest and farmland alongside roads and villages. Approximately 83 HAT cases were identified by the National Control Program in this focus between 1998 and 2011 (Eboʼo Eyenga, personal communication). In addition to HAT cases that involve *G. palpalis gambiensis* and *Tbg*, research in both foci has identified the presence of several other *Glossina*, including *G. p. palpalis* (*Gpp*), and *Trypanosoma* species, including *Trypanosoma congolense* (*Tc*). These previous studies also identified a variety of domestic and wild animals serving as reservoirs for diverse *Trypanosoma* species [44, 45, 58, 59]. As described in the following section, flies were trapped in these areas. Two tsetse fly trapping campaigns were conducted, one in May 2015 in three Campo villages (Ipono, Mabiogo and Campo-Beach), and the other in June 2015 in three Bipindi villages (Lambi, Bidjouka and Ebiminbang). The geographical positions of the sampling sites were determined by GPS.

### Fly sampling, dissection and DNA storage

Tsetse flies were captured using pyramidal traps according to Lancien [60], which were placed in suitable tsetse fly biotopes. Each trap was in place for four consecutive days, and flies were collected twice per day.

Sample processing included several steps, beginning with fly species identification on the basis of morphological criteria, using adapted taxonomic keys [38]. Non-teneral flies (flies that have taken a blood meal and thus may have become trypanosome-positive after having taken a meal on an infected host) were surface-sterilized, once with 5% sodium hypochlorite for 10 min and twice with 70% ethanol, each for 10 min. The whole gut of each fly was then dissected in a drop of sterile 0.9% saline solution according to Penchenier & Itard [61] under sterile conditions. The instruments used were carefully cleaned after the dissection of each fly to prevent cross-contamination. Guts were recovered and then separately transferred into tubes containing RNAlater (Ambion, Carlsbad, USA) for further DNA extraction and subsequent parasite identification using specific PCR amplification. Tubes containing the organs were stored at -20 °C for 5 days during field manipulations, and were subsequently stored in the laboratory at -80 °C until further processing.

### DNA extraction

Whole guts stored at -80 °C were thawed and RNAlater was removed by pipetting. In order to extract genomic DNA, guts were treated with a NucleoSpin TriPrep extraction kit (Macherey-Nagel, Hoerdt, France) according to the manufacturer’s instructions. One hundred microliters of DNA Elute solution was used to recover extracted DNA for each sample. DNA quantity and quality was inspected using a NanoDrop 2000 spectrophotometer (Thermo Fisher Scientific, Paris, France). All DNA samples were stored at -80 °C until use.

### Parasite identification by PCR amplification

The previously isolated DNA samples stored at -80 °C were thawed and used as templates for PCR amplification of highly repetitive satellite DNA sequences, as described by Sloof et al. [62]. Specific primers were used (Additional file 7: Table S3) that enabled identifying the trypanosome species that had infected the sampled tsetse flies.

PCR amplification of parasites was performed as previously described [63]. Specifically, the program included a denaturation step at 94 °C for 5 min, followed by 44 amplification cycles. Each cycle consisted of a denaturation step at 94 °C for 30 s, annealing at 55 °C for 30 s and an extension step at 72 °C for 1 min. A final extension step was performed at 72 °C for 10 min. The amplified products were separated on a 2% agarose gel containing ethidium bromide and visualized under UV illumination. Positive (2 ng of reference DNA) and negative controls were included in each PCR amplification experiment. PCR amplifications yielding a positive result were repeated once for confirmation.

### Sequencing by Illumina MiSeq

The amplicon sequencing approach was performed on a total of 190 individual DNA samples, using the Illumina MiSeq system (Illumina, San Diego, USA). Negative controls were processed similarly but without DNA. The primers and linkers used in this study are published elsewhere [55]; however, the primer design is described below. The primers were first constructed as described in Additional file 8: Figure S5, to which we added the appropriate sequencing primer read for Illumina MiSeq and the linker at the 5’ end of each specific primer. After primer synthesis, the *16S* rRNA gene was amplified for the V4 and V3V4 regions using V4F (5′-GTG TGC CAG CMG CCG CGG TAA-3′) and V4R (5′-CCG GAC TAC HVG GGT WTC TAA T-3′); and V3F (5′-GGC CTA CGG GAG GCA GCA G-3′) and V4R (5′-CCG GAC TAC HVG GGT WTC TAA T-3’), respectively. The V3V4 and V4 regions of the *16S* rRNA gene were PCR amplified using the respective forward primers 341F and 515F, along with the reverse primer 806R (for both cases). Amplicons were generated using a Diamont Taq® polymerase (Eurogentec, Paris, France). Amplicon lengths were 250 and 430 bp for the V4 and V3V4 regions, respectively.

PCR reactions were performed using the following cycling conditions: an initial denaturation at 95 °C for 2 min, 30 cycles at 95 °C for 20 s, 55 °C for 15 s and 72 °C for 5 min, and a final extension at 72 °C for 10 min. The expected sizes of the PCR products were verified on a 2% (w/v) agarose gel stained with ethidium bromide. The PCR products for both regions (V4 and V3V4) were then pooled in equimolar concentrations, and 4 µl was used in the second PCR step (Additional file 8: Figure S5) for multiplexing with indices i5 and i7. The P5 and P7 adapter sequences, indices and the sequencing primer partial site used to allow annealing of the amplicons to the flow cell were provided in the Illumina Nextera kit (Illumina) (http://umr-agap.cirad.fr/en/platforms/plateformes/genotyping).

Each reaction consisted of Master Mix 2×, the pooled PCR1 and water, with the following cycling conditions: an initial denaturation at 95 °C for 30 s, 5 cycles at 95 °C for 10 s, 60 °C for 30 s and 72 °C for 30 s, and a final extension at 72 °C for 5 min. The expected sizes of the PCR products were verified on a 2% (w/v) agarose gel stained with ethidium bromide.

The pooled amplicon library was purified using the Wizard® PCR Preps DNA Purification System (Promega, Paris, France). Agilent High Sensitivity DNA Kit (Agilent, Paris, France) was then used for the separation, sizing and quantification of the dsDNA samples. The final concentration of the library was determined using a SYBR green quantitative PCR (qPCR) assay with primers specific to the Illumina adapters (KAPA BioSystems, Wilmington, MA, USA). Libraries were then mixed with Illumina-generated PhiX control libraries and denatured using fresh NaOH prior to loading on the Illumina MiSeq flow-cell using a 500-cycle reagent cartridge and 2 × 251 bp paired-end sequencing.

The generated sequences have been deposited in the EMBL-EBI (study accession number PRJEB25818; secondary study accession number ERP107775).

### *16S* rRNA sequence data processing and taxonomic assignment

Since the initial data were retrieved in a demultiplexed state, the first stage in the workflow consisted in running VSearch v.2.3. [64] in order to merge the forward and reverse reads of each sample. VSearch allows the comparison of nucleotide sequences (during chimera detection, dereplication, searching and clustering) implementing an extremely fast Needleman-Wunsch algorithm. The expected read lengths from the V4 and V3V4 regions were 250 and 430 bp, respectively. The dataset was then filtered in 2 groups based on read length, comprising reads either shorter or longer than 400 bp and respectively corresponding to the V4 and V3V4 regions. We simultaneously carried out the sequencing of the V4 and V3V4 regions in order to refine the description of the bacterial populations in field flies, and to compare these two regions. This also allowed us to estimate the most effective region for use in a metabarcoding study of tsetse flies. Shirmer et al. [65] showed that the choice of primers can have a significant impact on the source of bias and cause distinct patterns of errors; the authors observed a gradual increase of errors as the read length increases. V3-V4 also represented a less-overlapping primer set as compared to V4, which might influence the quality check steps.

Subsequently, the remaining steps in the workflow were applied to the two resulting datasets. VSearch was used again to successively conduct primer clipping, exclusion of sequences containing unknown bases, expected error rate calculation, and sample-level dereplication in both datasets. The remaining sequences were then pooled into a single FASTA file to allow VSearch to perform a global dereplication, after which clustering was performed using Swarm v.2.1.1 [66]. VSearch was then used to identify chimeric clusters.

CutAdapt v.1.8.1 [67] was used with the primers to extract the V4 and V3V4 reference sequences from the SILVA SSU database (release 128), thereby creating a specific reference file for each targeted region. The STAMPA (https://github.com/frederic-mahe/stampa) pipeline was then run for taxonomic assignment of representative OTU sequences. This generated an OTU table to which the following filters were applied in order to retain targeted taxa at genus level: elimination of clusters with a high expected error, elimination of small clusters observed in less than three samples (taxa must be present in at least 3 individuals), and elimination of clusters with an abundance lower than or equal to 0.001.

The used pipeline makes it possible to define OTUs not on an arbitrary threshold of clustering but *via* the identification of biological variants (Swarms) identified using the following algorithm: start from the most abundant sequence then agglomerate sequences that differ from only one base pair and have a lower abundance; continue to explore the amplicon-space in all directions until no more amplicons could be added, or until a valley is found (i.e. an increase of amplicons’ abundances); if a valley is found, exploration stops in that particular direction, which allows to distinguish very similar biological variants; finally, to reduce OTU-noise level, virtual amplicons are generated to graft small “satellite” OTUs onto larger OTUs.

### Statistical analysis

To ensure that all bacteria present in the intestines of the flies were identified, we performed a rarefaction analysis of bacterial flora for each sample using Calypso software v.8.13 [68]. A search for a possible difference between the different taxa and the following parameters (Campo *vs* Bipindi focus and positive *vs* Tc negative flies infection status) was performed using the same software under a permutational multivariate analysis of variance test (PERMANOVA).

We calculated the Shannon diversity index (H) as well as the evenness and richness of the bacteria within the flies (i.e. the number of different bacterial genera and their abundance in the different fly samples) with respect to their geographical origin or the absence or presence of trypanosomes using Calypso v.8.13. A *P*-value below the fixed threshold (0.05) would therefore mean that a significant difference exists between the different populations in terms of their bacterial diversity.

The search for potential taxonomic groups that can serve as biomarkers for different classes (genera associated with specific condition) was performed using Lefse [69] contained in Calypso v.8.13. Reported statistically significant taxonomic groups showed high linear discriminant analysis scores, which indicate the degree of consistency in relative abundance between taxonomic groups together with their effect relevance in each class.

The principal components analysis (PCA), using the Bray-Curtis index which takes into account the relative abundance of bacteria, was performed with Calypso v.8.13. The aim was to find out whether there was a differential aggregation of *Trypanosoma congolense* (*s.l.*) positive and non-positive tsetse flies between Campo and Bipindi tsetse flies. PCA was also performed using the Jaccard diversity index which take into account the presence/absence of bacteria.

Finally, to have a more detailed view of bacterial flora according to the parameters (focus of origin and infection status), a simple description of bacteria considering each parameter as a data set was done. The bacteria were described separately in Campo flies, Bipindi, in Tc-positive flies and finally in negative ones. Bacteria present in one condition and not in the other were considered as potential biomarkers of a condition (using a simple differential description). The description criteria were the same as above (taxa must be present in at least 3 individuals and with an abundance higher than 0.001).

## Additional files

**Additional file 1: Table S1.** Number of reads mapping to each individual OTU in the SILVA database for all individual flies. The V3V4 and V4 regions of the *16S* rRNA gene were PCR amplified using the respective forward and reverse primers. Amplicons were generated using a Diamont Taq® polymerase and amplicon lengths were 250 and 430 bp for the V4 and V3V4 regions, respectively. The used pipeline makes it possible to define individual OTU for each samples.

**Additional file 2: Figure S1.** Rarefaction curve for all sequenced samples. **a** Data derived from sequencing of the V3V4 region. **b** Data derived from sequencing of the V4 region. The red squares represent the controls that were introduced during the experiments to ensure the success of the sequencing. *Abbreviations*: F, female; M, male.

**Additional file 3: Figure S2.** Summary of bacterial abundances in different conditions. **a** Abundances with *Wigglesworthia*. **b** Abundances without *Wigglesworthia*. Infected: positive with *Trypanosoma congolense* (*s.l.*). *Abbreviation*: w_w, without *Wigglesworthia*.

**Additional file 4: Figure S3.** Principal components analysis (PCA) using the Bray-Curtis diversity index based on the focus origin (Campo or Bipindi) (**a**) or infection status (**b**). *Key*: Bipindi, flies harvested in Bipindi; Campo, flies harvested in Campo; TcF, *T. congolense* “forest” type; TcS, *T. congolense* “savannah” type; mixed infection, flies simultaneously positive by both parasites (TcF + TcS).

**Additional file 5: Table S2.** Non-normalized abundance table (number of reads per sample).

**Additional file 6: Figure S4.** Principal components analysis (PCA) using the Jaccard diversity index based on the focus origin (Campo or Bipindi) (**a**) or infection status (**b**). *Key*: Bipindi, flies harvested in Bipindi; Campo, flies harvested in Campo; TcF, *T. congolense* “forest” type; TcS, *T. congolense* “savannah” type; mixed infection, flies simultaneously positive for both parasites (TcF + TcS).

**Additional file 7: Table S3.** Primers used for PCR amplification of trypanosomes.

**Additional file 8: Figure S5.** Workflow of the amplicon library construction. The target region of interest from the Nextera kit is shown.

## Abbreviations

HAT: human African trypanosomiasis
AAT: African animal trypanosomiasis
WHO: World Health Organization
PATTEC: Pan-African Tsetse and Trypanosomiasis Eradication Campaign
*Tbg*: *Trypanosoma brucei gambiense*
*Tbr*: *T. b. rhodesiense*
*Tbb*: *T. b. brucei*
*Tc*: *T. congolense*
*Tv*: *T. vivax*
OTU: operational taxonomic unit
PERMANOVA: permutational multivariate analysis of variance
VSG: variable surface glycoprotein
PCA: principal components analysis
GPS: global postioning system
SILVA: ribosomal RNA database
SSU: small subunit
IAEA: International Atomic Energy Agency
