# Supplementary material for: The composition and abundance of bacterial communities residing in the gut of Glossina palpalis palpalis captured in two sites of southern Cameroon
Source: Parasit Vectors. 2019 Apr 2;12:151. doi: 10.1186/s13071-019-3402-2 (PMC6444424; doi:10.1186/s13071-019-3402-2)
Supplement: Supplementary file 1 — Additional file 1: Table S1. Number of reads mapping to each individual OTU in the SILVA database for all individual flies. The V3V4 and V4 regions of the 16S rRNA gene were PCR amplified using the respective forward and reverse primers. Amplicons were generated using a Diamont Taq® polymerase and amplicon lengths were 250 and 430 bp for the V4 and V3V4 regions, respectively. The used pipeline makes it possible to define individual OTU for each samples. [file 13071_2019_3402_MOESM1_ESM.pdf]

Table S1a: number of reads (V4 region) mapping to each individual OTU in the SILVA database for all individual flies

Identity= the best hits of number of reads mapping to each individual OTU in the SILVA database

genus	Wigglesworthia	Salmonella	Spiroplasma	Sphingomonas	Uncultured bacterium	Methylobacterium
total	4227129	34224	2370	1802	909	516
identity	97.94	100	100	100	95	100
J1-10_S76	809	0	0	34	1	0
J1-11_S112	16300	0	5	1	0	0
J1-21_S88	16669	0	0	16	2	3
J1-23_S94	22786	0	73	4	0	2
J1-28_S14	27999	0	0	25	1	9
J1-31_S49	39896	0	123	5	0	5
J100-7_S38	59274	0	0	8	0	0
J11-6_S63	21381	0	0	7	0	3
J11-7_S71	7	0	0	3	0	3
J12-1_S56	17482	0	0	48	1	5
J15-4_S58	54733	0	0	44	1	19
J15-5_S119	415	0	1	12	0	0
J16-13_S103	22445	0	0	2	0	0
J16-14_S55	19417	0	0	20	1	2
J16-16_S39	1816	0	0	13	0	5
J16-1_S168	243	0	0	23	0	2
J16-23_S69	31895	0	0	13	2	1
J16-33_S158	23704	0	0	1	0	0
J16-34_S160	6763	0	0	2	0	0
J16-35_S75	20073	0	0	6	0	6
J16-37_S189	21890	0	0	11	0	0
J16-40_S64	94747	0	42	15	1	11
J16-41_S102	9526	0	10	6	0	0
J16-43_S21	5727	0	0	7	0	13
J16-45_S82	16203	0	0	24	19	9
J16-51_S50	65869	1	169	40	6	4
J16-53_S169	10706	4	0	4	0	0
J17-11_S24	3067	0	0	3	2	2
J17-20_S120	21031	0	9	0	0	0
J17-23_S81	20343	0	0	30	8	6
J17-27_S142	34042	482	0	9	0	0
J17-42_S12	14571	0	0	26	0	7
J17-6_S20	31353	0	0	7	0	13
J18-10_S40	1997	0	0	49	2	20
J18-11_S42	2940	0	4	7	0	10
J18-13_S148	21028	0	11	3	3	0
J18-14_S99	14300	0	0	0	0	5
J18-15_S166	45561	0	22	4	0	2
J18-16_S35	3833	0	116	10	129	4
J18-17_S72	17752	0	0	15	0	4
J18-18_S77	10382	0	0	5	0	1

J18-18bis_S179	7375	0	0	1	0	4
J18-24_S111	18218	0	19	5	1	1
J18-25_S184	16520	0	0	1	0	0
J18-27_S122	17528	0	0	2	0	0
J18-34_S26	21880	0	0	6	3	6
J18-37_S53	69436	0	0	8	0	1
J18-38_S147	22966	0	3	4	2	0
J18-45_S175	13474	0	8	2	0	0
J18-9_S136	9957	0	0	4	0	0
J19-10_S87	10126	0	0	10	69	0
J19-17_S127	15382	0	9	7	0	1
J19-1_S157	13215	0	0	0	0	1
J19-20_S9	36150	1	0	35	0	0
J19-22_S182	9445	0	0	9	0	3
J2-5_S18	39434	0	0	8	0	2
J2-6_S186	4553	0	0	1	1	9
J2-7_S22	22111	0	0	7	0	1
J2-8_S151	11085	0	0	8	0	0
J20-10_S66	34678	0	0	11	0	0
J20-25_S30	31035	0	116	15	0	3
J20-27_S98	1430	0	0	0	0	0
J20-31_S29	29015	0	0	1	0	0
J20-34_S59	26968	1	0	5	2	0
J20-4_S95	164	0	0	32	12	1
J20-4bis_S101	149	0	0	0	0	2
J20-7_S45	20844	0	0	27	2	1
J20-9_S44	38676	0	0	11	3	4
J21-10_S37	56879	0	0	11	0	0
J22-13_S130	4513	0	0	10	0	2
J22-1_S183	6229	0	0	0	0	0
J22-2_S152	3338	0	0	10	0	0
J22-7_S143	21113	0	17	2	1	0
J23-6_S92	9333	0	0	13	2	1
J23-7_S114	20876	0	0	2	0	0
J24-10_S36	19544	0	23	6	11	0
J25-3_S164	48043	0	19	12	1	12
J26-1_S17	18153	0	0	11	0	2
J26-2_S128	13996	0	0	2	0	1
J27-13_S190	31006	1	0	4	0	0
J28-10_S150	2333	0	0	1	1	0
J28-5_S149	7294	0	0	9	0	4
J28-9_S91	12691	0	0	3	0	0
J29-16_S80	8488	0	9	15	0	1
J29-17_S61	9938	0	0	29	0	3
J29-17bis_S131	8318	0	0	1	1	1
J3-4_S181	16377	0	63	2	3	1
J30-19_S170	54958	0	0	0	0	0
J30-6_S165	9993	0	0	7	1	4
J30-8_S133	3058	0	10	23	0	0
J30-9_S83	14382	0	0	5	0	5

J31-15_S16	38516	0	0	7	2	1
J31-15bis_S104	17529	0	0	7	0	3
J31-18_S1	46586	0	0	14	546	0
J31-25_S6	38884	0	0	6	2	6
J31-26_S138	26292	0	0	1	0	9
J32-2_S25	23436	0	42	11	0	21
J32-5_S153	29938	464	0	9	0	2
J33-13_S100	13548	0	0	1	0	6
J34-16_S106	26473	0	0	3	0	0
J34-2_S47	17896	0	0	8	0	1
J34-2bis_S118	25049	0	0	11	1	5
J34-3_S33	67066	0	0	16	1	4
J34-4_S46	9661	0	0	24	4	11
J35-15_S15	30992	0	0	10	0	2
J35-8_S129	16476	0	0	8	6	0
J35-9_S74	18957	0	0	12	2	2
J36-13_S154	18349	0	0	3	0	0
J36-1_S105	25023	0	0	3	2	1
J36-3_S173	11934	0	0	0	0	2
J37-1_S115	38915	0	16	1	0	2
J37-1bis_S62	110312	0	55	13	1	0
J38-1_S134	5102	0	23	0	0	0
J38-3_S51	59617	0	0	18	0	5
J39-4_S161	24287	0	0	0	0	1
J39-6_S68	12390	0	0	37	1	4
J4-10_S176	35515	0	32	2	2	0
J4-16_S31	16761	0	0	23	0	0
J4-17_S32	32127	0	6	16	0	4
J4-25_S146	7702	0	0	6	1	0
J4-32_S121	551	0	0	4	0	3
J40-10_S178	4052	0	0	0	0	2
J40-11_S135	4385	0	21	4	2	0
J40-14_S28	25964	0	0	18	0	0
J40-1a_S34	267	0	0	8	0	4
J40-1b_S97	6394	0	6	1	1	0
J40-2_S79	6501	0	0	35	1	4
J40-4_S163	11296	0	0	4	0	0
J40-7_S162	47002	0	0	6	0	7
J41-5_S93	15769	1430	0	2	0	3
J41-8_S167	15236	0	0	2	0	2
J42-4_S132	5619	0	0	1	0	0
J42-6_S113	9938	0	6	4	0	0
J45-3_S117	19055	0	0	6	1	0
J46-3_S23	35944	0	0	2	0	0
J47-12_S84	27149	0	0	5	0	1
J47-16_S13	41415	0	0	1	1	0
J47-17_S43	19339	0	434	16	0	5
J47-20_S174	50964	0	91	8	0	1
J47-23_S159	13260	0	15	2	0	0
J47-2_S116	16966	0	0	3	0	13

J47-2bis_S48	29735	0	0	25	0	15
J47-31_S137	43043	2	0	3	0	0
J47-6_S4	41395	0	0	4	0	3
J47-9_S78	27558	0	0	10	0	1
J47-9bis_S180	24602	1	0	0	0	0
J48-4_S3	43774	0	0	8	0	2
J52-14_S171	19161	0	0	3	0	0
J52-6_S124	20423	0	0	0	0	0
J55-14_S5	38259	0	28	12	2	7
J55-43_S86	20676	0	0	9	0	2
J56-1_S177	3078	0	0	6	0	2
J58-1_S187	2411	0	0	4	0	1
J58-24_S140	28287	0	0	5	1	8
J58-2_S185	33748	31822	0	2	0	0
J59-4_S73	24584	0	0	9	0	1
J6-2_S144	25273	0	56	1	2	3
J6-3_S27	961	0	0	10	0	2
J6-5_S145	30975	0	0	1	0	0
J60-18_S172	45988	0	206	14	2	1
J65-36_S110	30577	1	49	0	0	1
J68-3_S139	2948	0	6	2	1	0
J68-9_S126	21622	0	21	3	0	1
J7-1_S57	6002	6	0	41	0	11
J7-1bis_S191	9880	0	0	3	0	0
J7-9_S89	23723	0	20	5	0	0
J70-8_S107	10808	0	0	14	0	0
J73-49_S125	25895	1	0	0	0	0
J73-51_S123	16717	0	0	4	0	3
J73-68_S7	22761	0	144	11	5	1
J77-12_S8	16824	3	0	13	2	7
J78-56_S70	27363	0	0	14	0	2
J79-43_S156	8409	0	0	5	0	0
J79-98_S65	39059	0	159	11	1	3
J79-99_S67	36661	0	0	7	0	8
J8-5_S10	23726	0	34	13	1	4
J8-6_S11	11618	0	0	13	2	0
J8-9_S60	21642	0	0	54	17	6
J80-115_S52	82340	0	0	12	0	9
J80-116_S155	25209	0	0	3	0	0
J80-117_S54	27970	1	0	1	0	0
J83-3_S108	22823	0	0	2	0	4
J84-21_S85	28453	0	0	0	0	0
J88-1_S109	18258	0	0	5	1	1
J89-8_S141	32	1	0	3	0	1
J9-6_S41	28293	1	19	7	0	0
J9-8_S90	20264	0	0	23	0	10
J9-9_S2	18670	0	0	1	0	0
J94-4_S188	35329	1	0	1	0	0
J95-11_S19	22990	0	0	18	0	4
NTC-1_S96	10	0	0	17	0	2

NTC-2_S192	281	0	0	8	0	0

genus	Acidibacter	Tsukamurella	Serratia
total	94	91	53
identity	100	100	98
J1-10_S76	1	0	0
J1-11_S112	0	0	0
J1-21_S88	0	0	0
J1-23_S94	0	0	0
J1-28_S14	0	0	0
J1-31_S49	0	0	0
J100-7_S38	0	0	0
J11-6_S63	0	0	0
J11-7_S71	0	0	0
J12-1_S56	1	0	0
J15-4_S58	8	0	0
J15-5_S119	0	0	0
J16-13_S103	2	0	0
J16-14_S55	0	0	0
J16-16_S39	1	0	0
J16-1_S168	0	0	0
J16-23_S69	0	1	0
J16-33_S158	0	0	0
J16-34_S160	0	0	0
J16-35_S75	0	0	0
J16-37_S189	0	0	0
J16-40_S64	0	0	0
J16-41_S102	0	0	0
J16-43_S21	0	0	0
J16-45_S82	0	0	0
J16-51_S50	0	0	0
J16-53_S169	0	0	0
J17-11_S24	0	0	0
J17-20_S120	0	0	0
J17-23_S81	0	0	0
J17-27_S142	0	0	0
J17-42_S12	0	0	0
J17-6_S20	0	0	0
J18-10_S40	0	0	0
J18-11_S42	0	0	0
J18-13_S148	0	0	0
J18-14_S99	5	0	0
J18-15_S166	4	0	0
J18-16_S35	0	0	0
J18-17_S72	0	0	0
J18-18_S77	0	0	0
J18-18bis_S179	0	0	0

J18-24_S111	0	0	0
J18-25_S184	2	0	0
J18-27_S122	0	0	0
J18-34_S26	0	0	0
J18-37_S53	0	0	0
J18-38_S147	0	0	0
J18-45_S175	1	0	0
J18-9_S136	0	0	0
J19-10_S87	1	0	0
J19-17_S127	3	0	0
J19-1_S157	0	0	0
J19-20_S9	1	0	0
J19-22_S182	2	0	0
J2-5_S18	0	0	0
J2-6_S186	0	0	1
J2-7_S22	0	0	0
J2-8_S151	0	0	0
J20-10_S66	0	0	0
J20-25_S30	0	0	0
J20-27_S98	0	0	0
J20-31_S29	0	0	0
J20-34_S59	0	0	0
J20-4_S95	0	0	0
J20-4bis_S101	0	0	0
J20-7_S45	0	0	0
J20-9_S44	0	0	0
J21-10_S37	0	0	0
J22-13_S130	0	0	0
J22-1_S183	5	0	0
J22-2_S152	4	0	0
J22-7_S143	0	0	0
J23-6_S92	0	0	0
J23-7_S114	0	0	0
J24-10_S36	0	0	0
J25-3_S164	1	0	0
J26-1_S17	0	0	0
J26-2_S128	0	0	0
J27-13_S190	1	0	0
J28-10_S150	5	0	0
J28-5_S149	2	0	0
J28-9_S91	0	0	0
J29-16_S80	0	0	0
J29-17_S61	7	0	0
J29-17bis_S131	0	0	0
J3-4_S181	0	0	0
J30-19_S170	2	0	0
J30-6_S165	0	0	0
J30-8_S133	0	0	0
J30-9_S83	0	0	0
J31-15_S16	0	0	0

J31-15bis_S104	0	0	0
J31-18_S1	0	87	0
J31-25_S6	0	0	0
J31-26_S138	0	0	0
J32-2_S25	0	0	0
J32-5_S153	0	0	0
J33-13_S100	0	0	0
J34-16_S106	0	0	0
J34-2_S47	0	0	0
J34-2bis_S118	0	0	0
J34-3_S33	0	0	0
J34-4_S46	0	0	0
J35-15_S15	0	0	0
J35-8_S129	0	0	0
J35-9_S74	0	0	0
J36-13_S154	0	2	0
J36-1_S105	0	0	0
J36-3_S173	0	0	0
J37-1_S115	0	0	0
J37-1bis_S62	0	0	0
J38-1_S134	2	0	0
J38-3_S51	0	0	0
J39-4_S161	0	0	0
J39-6_S68	4	0	0
J4-10_S176	0	0	0
J4-16_S31	1	0	0
J4-17_S32	0	0	0
J4-25_S146	0	0	0
J4-32_S121	0	0	0
J40-10_S178	0	0	0
J40-11_S135	2	0	0
J40-14_S28	0	0	0
J40-1a_S34	0	0	0
J40-1b_S97	0	0	0
J40-2_S79	0	0	0
J40-4_S163	0	0	0
J40-7_S162	0	0	0
J41-5_S93	0	0	11
J41-8_S167	1	0	0
J42-4_S132	0	0	0
J42-6_S113	0	0	0
J45-3_S117	0	0	0
J46-3_S23	0	0	0
J47-12_S84	0	0	0
J47-16_S13	0	0	0
J47-17_S43	3	0	0
J47-20_S174	0	0	0
J47-23_S159	0	0	0
J47-2_S116	0	0	0
J47-2bis_S48	0	0	0

J47-31_S137	0	1	0
J47-6_S4	0	0	0
J47-9_S78	0	0	0
J47-9bis_S180	2	0	0
J48-4_S3	1	0	0
J52-14_S171	0	0	0
J52-6_S124	0	0	0
J55-14_S5	1	0	0
J55-43_S86	2	0	0
J56-1_S177	0	0	0
J58-1_S187	0	0	0
J58-24_S140	0	0	0
J58-2_S185	2	0	41
J59-4_S73	0	0	0
J6-2_S144	0	0	0
J6-3_S27	1	0	0
J6-5_S145	0	0	0
J60-18_S172	0	0	0
J65-36_S110	0	0	0
J68-3_S139	0	0	0
J68-9_S126	0	0	0
J7-1_S57	1	0	0
J7-1bis_S191	0	0	0
J7-9_S89	0	0	0
J70-8_S107	4	0	0
J73-49_S125	0	0	0
J73-51_S123	0	0	0
J73-68_S7	0	0	0
J77-12_S8	0	0	0
J78-56_S70	0	0	0
J79-43_S156	0	0	0
J79-98_S65	2	0	0
J79-99_S67	0	0	0
J8-5_S10	2	0	0
J8-6_S11	0	0	0
J8-9_S60	0	0	0
J80-115_S52	0	0	0
J80-116_S155	0	0	0
J80-117_S54	0	0	0
J83-3_S108	3	0	0
J84-21_S85	0	0	0
J88-1_S109	0	0	0
J89-8_S141	1	0	0
J9-6_S41	0	0	0
J9-8_S90	0	0	0
J9-9_S2	0	0	0
J94-4_S188	0	0	0
J95-11_S19	0	0	0
NTC-1_S96	0	0	0
NTC-2_S192	0	0	0

Table S1b: number of reads (V3V4 region) mapping to each individual OTU in the SILVA database for all individual flies

Identity= the best hits of number of reads mapping to each individual OTU in the SILVA database

Genus	Wigglesworthia	Uncultured bacterium	Serratia	Kluyvera	Spiroplasma	Sphingomonas
Total	1578889	11211	3494	2769	808	690
Identity	96.28	91.7	99.76	99.75	100	100
J1-10_S76	183	0	0	0	0	7
J1-11_S112	8995	0	0	0	2	0
J1-21_S88	3996	0	0	0	0	10
J1-23_S94	8330	0	0	0	20	2
J1-28_S14	13561	0	0	0	0	2
J1-31_S49	13105	0	0	0	54	4
J100-7_S38	17800	0	0	0	0	3
J11-6_S63	3409	0	0	0	0	4
J11-7_S71	7	0	0	0	0	12
J12-1_S56	4219	0	0	0	0	29
J15-4_S58	12386	0	0	0	0	4
J15-5_S119	158	0	0	0	4	0
J16-13_S103	14473	0	0	0	0	1
J16-14_S55	4126	0	0	0	0	4
J16-16_S39	465	0	0	0	0	5
J16-1_S168	172	0	0	0	0	3
J16-23_S69	11052	0	0	0	0	10
J16-33_S158	12266	0	0	0	0	2
J16-34_S160	3581	0	0	0	0	3
J16-35_S75	4718	0	0	0	0	4
J16-37_S189	3825	0	0	0	0	2
J16-40_S64	24113	0	0	0	4	19
J16-41_S102	6035	0	0	0	7	0
J16-43_S21	1450	0	0	0	0	6
J16-45_S82	2369	0	0	0	0	1
J16-51_S50	15286	0	0	0	22	18
J16-53_S169	9077	0	0	0	0	3
J17-11_S24	892	0	0	0	0	3
J17-20_S120	11004	0	0	0	0	0
J17-23_S81	4191	0	0	0	0	2
J17-27_S142	17272	307	0	0	0	1
J17-42_S12	4070	0	0	0	0	7
J17-6_S20	7299	0	0	0	0	6
J18-10_S40	570	0	0	0	0	3
J18-11_S42	490	0	0	0	2	12
J18-13_S148	9994	0	0	0	21	4
J18-14_S99	9927	0	0	0	0	0
J18-15_S166	27647	0	0	0	30	2
J18-16_S35	1319	0	0	0	30	4

J18-17_S72	5129	0	0	0	0	6
J18-18_S77	2972	0	0	0	0	2
J18-18bis_S179	10565	0	0	0	0	4
J18-24_S111	6978	0	0	0	8	2
J18-25_S184	9191	0	0	0	0	3
J18-27_S122	11978	0	0	0	0	3
J18-34_S26	3606	0	0	0	0	6
J18-37_S53	13057	0	0	0	0	2
J18-38_S147	13784	0	0	0	8	1
J18-45_S175	13462	0	0	0	9	7
J18-9_S136	5448	0	0	0	0	1
J19-10_S87	4063	0	0	0	0	6
J19-17_S127	6469	0	0	0	8	2
J19-1_S157	6099	0	0	0	0	1
J19-20_S9	8629	0	0	0	0	1
J19-22_S182	5073	0	0	0	0	4
J2-5_S18	14455	0	0	0	0	3
J2-6_S186	2583	0	0	0	0	2
J2-7_S22	11107	0	0	0	0	4
J2-8_S151	9079	0	0	0	0	2
J20-10_S66	5828	0	0	0	0	0
J20-25_S30	7975	0	0	0	14	8
J20-27_S98	9591	0	0	0	0	3
J20-31_S29	11228	0	0	0	0	0
J20-34_S59	3360	0	0	0	0	2
J20-4_S95	62	0	0	0	0	27
J20-4bis_S101	56	0	0	0	0	7
J20-7_S45	3995	0	0	0	0	4
J20-9_S44	8885	0	0	0	0	2
J21-10_S37	16042	0	0	0	0	1
J22-13_S130	3787	0	0	0	0	0
J22-1_S183	3567	0	0	0	0	2
J22-2_S152	2217	0	0	0	0	1
J22-7_S143	12884	0	0	0	6	2
J23-6_S92	2990	0	0	0	0	2
J23-7_S114	13521	0	0	0	0	1
J24-10_S36	3237	0	0	0	7	0
J25-3_S164	26607	0	0	0	18	6
J26-1_S17	1639	0	0	0	0	10
J26-2_S128	11218	0	0	0	0	0
J27-13_S190	14335	0	0	0	0	1
J28-10_S150	1412	0	0	0	0	1
J28-5_S149	4231	0	0	0	0	7
J28-9_S91	4405	0	0	0	0	0
J29-16_S80	5878	0	0	0	6	14
J29-17_S61	1271	1	0	0	0	7
J29-17bis_S131	3610	0	0	0	0	2
J3-4_S181	12022	0	0	0	28	23
J30-19_S170	37903	0	0	0	0	0
J30-6_S165	7487	0	0	0	0	4

J30-8_S133	1582	0	0	0	19	12
J30-9_S83	3605	0	0	0	0	3
J31-15_S16	7298	0	0	0	0	2
J31-15bis_S104	7733	0	0	0	0	0
J31-18_S1	1438	0	0	0	0	0
J31-25_S6	9759	0	0	0	0	1
J31-26_S138	15451	0	0	0	0	0
J32-2_S25	7555	0	0	0	14	2
J32-5_S153	24619	154	50	38	0	1
J33-13_S100	15219	0	0	0	0	0
J34-16_S106	13770	0	0	0	0	2
J34-2_S47	3711	0	0	0	0	2
J34-2bis_S118	10732	0	0	0	0	3
J34-3_S33	11117	0	0	0	0	6
J34-4_S46	2819	0	0	0	0	4
J35-15_S15	6890	0	0	0	0	4
J35-8_S129	11315	0	0	0	0	0
J35-9_S74	5614	0	0	0	0	3
J36-13_S154	9568	0	0	0	0	3
J36-1_S105	14765	0	0	0	0	1
J36-3_S173	10010	0	0	0	0	3
J37-1_S115	8532	0	0	0	7	0
J37-1bis_S62	30765	0	0	0	17	9
J38-1_S134	3850	0	0	0	13	4
J38-3_S51	5553	0	0	0	0	0
J39-4_S161	18998	0	0	0	0	0
J39-6_S68	3142	0	0	0	0	1
J4-10_S176	15053	0	0	0	12	1
J4-16_S31	3920	0	0	0	0	2
J4-17_S32	8686	0	0	0	0	11
J4-25_S146	4473	0	0	0	0	0
J4-32_S121	155	0	0	0	0	2
J40-10_S178	5007	0	0	0	0	4
J40-11_S135	2351	0	0	0	5	0
J40-14_S28	7644	0	0	0	0	16
J40-1a_S34	53	0	0	0	0	5
J40-1b_S97	10932	0	0	0	9	0
J40-2_S79	1455	0	0	0	0	6
J40-4_S163	10591	0	0	0	0	1
J40-7_S162	27215	0	0	0	0	5
J41-5_S93	5435	206	228	0	0	0
J41-8_S167	13065	0	0	0	0	2
J42-4_S132	6863	0	0	0	0	0
J42-6_S113	2678	0	0	0	2	0
J45-3_S117	8814	0	0	0	0	3
J46-3_S23	13170	0	0	0	0	1
J47-12_S84	9019	0	0	0	0	3
J47-16_S13	9975	0	0	0	0	0
J47-17_S43	3642	0	0	0	80	2
J47-20_S174	11954	0	0	0	12	1

J47-23_S159	7541	0	0	0	6	0
J47-2_S116	7224	0	0	0	0	0
J47-2bis_S48	7563	0	0	0	0	8
J47-31_S137	28124	0	0	0	0	0
J47-6_S4	7526	0	0	0	0	3
J47-9_S78	7883	0	0	0	0	1
J47-9bis_S180	14838	0	0	0	0	3
J48-4_S3	12025	0	0	0	0	6
J52-14_S171	10984	0	0	0	0	0
J52-6_S124	13001	0	0	0	0	2
J55-14_S5	8588	0	0	0	2	2
J55-43_S86	7050	0	0	0	0	7
J56-1_S177	2480	1	0	0	0	0
J58-1_S187	1296	0	0	0	0	0
J58-24_S140	8628	0	0	0	0	2
J58-2_S185	833	10541	3216	2730	0	0
J59-4_S73	9146	0	0	0	0	1
J6-2_S144	14507	0	0	0	26	0
J6-3_S27	316	0	0	0	0	8
J6-5_S145	17844	0	0	0	0	0
J60-18_S172	27903	0	0	0	137	4
J65-36_S110	10813	0	0	0	17	0
J68-3_S139	2320	0	0	0	8	1
J68-9_S126	13343	0	0	0	13	0
J7-1_S57	1066	1	0	0	0	13
J7-1bis_S191	6185	0	0	0	0	0
J7-9_S89	9460	0	0	0	13	0
J70-8_S107	3065	0	0	0	0	0
J73-49_S125	9328	0	0	0	0	1
J73-51_S123	6298	0	0	0	0	1
J73-68_S7	7335	0	0	0	55	13
J77-12_S8	5316	0	0	0	0	5
J78-56_S70	6673	0	0	0	0	6
J79-43_S156	5779	0	0	0	0	1
J79-98_S65	2603	0	0	0	10	1
J79-99_S67	9974	0	0	0	0	9
J8-5_S10	6030	0	0	0	18	4
J8-6_S11	3487	0	0	0	0	4
J8-9_S60	6043	0	0	0	0	6
J80-115_S52	25498	0	0	0	0	3
J80-116_S155	12874	0	0	0	0	0
J80-117_S54	9840	0	0	0	0	0
J83-3_S108	9812	0	0	0	0	0
J84-21_S85	11764	0	0	0	0	3
J88-1_S109	8324	0	0	0	0	2
J89-8_S141	6	0	0	0	0	0
J9-6_S41	9545	0	0	1	5	13
J9-8_S90	3984	0	0	0	0	4
J9-9_S2	6933	0	0	0	0	9
J94-4_S188	1385	0	0	0	0	0

J95-11_S19	2144	0	0	0	0	12

Genus	Methylobacterium
Total	248
Identity	100
J1-10_S76	3
J1-11_S112	0
J1-21_S88	0
J1-23_S94	0
J1-28_S14	5
J1-31_S49	4
J100-7_S38	1
J11-6_S63	0
J11-7_S71	0
J12-1_S56	16
J15-4_S58	9
J15-5_S119	10
J16-13_S103	1
J16-14_S55	1
J16-16_S39	1
J16-1_S168	0
J16-23_S69	2
J16-33_S158	0
J16-34_S160	0
J16-35_S75	0
J16-37_S189	0
J16-40_S64	1
J16-41_S102	1
J16-43_S21	5
J16-45_S82	5
J16-51_S50	1
J16-53_S169	0
J17-11_S24	2
J17-20_S120	0
J17-23_S81	2
J17-27_S142	0
J17-42_S12	1
J17-6_S20	0
J18-10_S40	0
J18-11_S42	2
J18-13_S148	0
J18-14_S99	0
J18-15_S166	0
J18-16_S35	5
J18-17_S72	0
J18-18_S77	1
J18-18bis_S179	1

J18-24_S111	0
J18-25_S184	0
J18-27_S122	0
J18-34_S26	2
J18-37_S53	0
J18-38_S147	2
J18-45_S175	0
J18-9_S136	2
J19-10_S87	0
J19-17_S127	0
J19-1_S157	0
J19-20_S9	0
J19-22_S182	0
J2-5_S18	2
J2-6_S186	1
J2-7_S22	3
J2-8_S151	0
J20-10_S66	2
J20-25_S30	3
J20-27_S98	1
J20-31_S29	0
J20-34_S59	0
J20-4_S95	2
J20-4bis_S101	4
J20-7_S45	1
J20-9_S44	5
J21-10_S37	3
J22-13_S130	0
J22-1_S183	0
J22-2_S152	0
J22-7_S143	0
J23-6_S92	0
J23-7_S114	1
J24-10_S36	0
J25-3_S164	10
J26-1_S17	0
J26-2_S128	0
J27-13_S190	0
J28-10_S150	0
J28-5_S149	0
J28-9_S91	0
J29-16_S80	2
J29-17_S61	0
J29-17bis_S131	0
J3-4_S181	3
J30-19_S170	2
J30-6_S165	0
J30-8_S133	0
J30-9_S83	2
J31-15_S16	0

J31-15bis_S104	0
J31-18_S1	0
J31-25_S6	0
J31-26_S138	0
J32-2_S25	3
J32-5_S153	2
J33-13_S100	2
J34-16_S106	0
J34-2_S47	2
J34-2bis_S118	2
J34-3_S33	0
J34-4_S46	4
J35-15_S15	4
J35-8_S129	0
J35-9_S74	1
J36-13_S154	0
J36-1_S105	0
J36-3_S173	0
J37-1_S115	0
J37-1bis_S62	1
J38-1_S134	0
J38-3_S51	0
J39-4_S161	0
J39-6_S68	2
J4-10_S176	1
J4-16_S31	1
J4-17_S32	7
J4-25_S146	2
J4-32_S121	5
J40-10_S178	0
J40-11_S135	1
J40-14_S28	0
J40-1a_S34	2
J40-1b_S97	0
J40-2_S79	6
J40-4_S163	0
J40-7_S162	0
J41-5_S93	2
J41-8_S167	2
J42-4_S132	2
J42-6_S113	0
J45-3_S117	2
J46-3_S23	0
J47-12_S84	0
J47-16_S13	0
J47-17_S43	0
J47-20_S174	1
J47-23_S159	0
J47-2_S116	3
J47-2bis_S48	1

J47-31_S137	0
J47-6_S4	0
J47-9_S78	0
J47-9bis_S180	1
J48-4_S3	0
J52-14_S171	0
J52-6_S124	1
J55-14_S5	0
J55-43_S86	3
J56-1_S177	0
J58-1_S187	0
J58-24_S140	0
J58-2_S185	0
J59-4_S73	0
J6-2_S144	3
J6-3_S27	3
J6-5_S145	0
J60-18_S172	0
J65-36_S110	0
J68-3_S139	0
J68-9_S126	0
J7-1_S57	3
J7-1bis_S191	2
J7-9_S89	0
J70-8_S107	5
J73-49_S125	0
J73-51_S123	0
J73-68_S7	1
J77-12_S8	0
J78-56_S70	0
J79-43_S156	0
J79-98_S65	0
J79-99_S67	0
J8-5_S10	8
J8-6_S11	4
J8-9_S60	3
J80-115_S52	0
J80-116_S155	1
J80-117_S54	0
J83-3_S108	0
J84-21_S85	0
J88-1_S109	0
J89-8_S141	0
J9-6_S41	2
J9-8_S90	1
J9-9_S2	0
J94-4_S188	0
J95-11_S19	13
