# Supplementary figures and images for: The composition and abundance of bacterial communities residing in the gut of Glossina palpalis palpalis captured in two sites of southern Cameroon
Source: Parasit Vectors. 2019 Apr 2;12:151. doi: 10.1186/s13071-019-3402-2 (PMC6444424; doi:10.1186/s13071-019-3402-2)

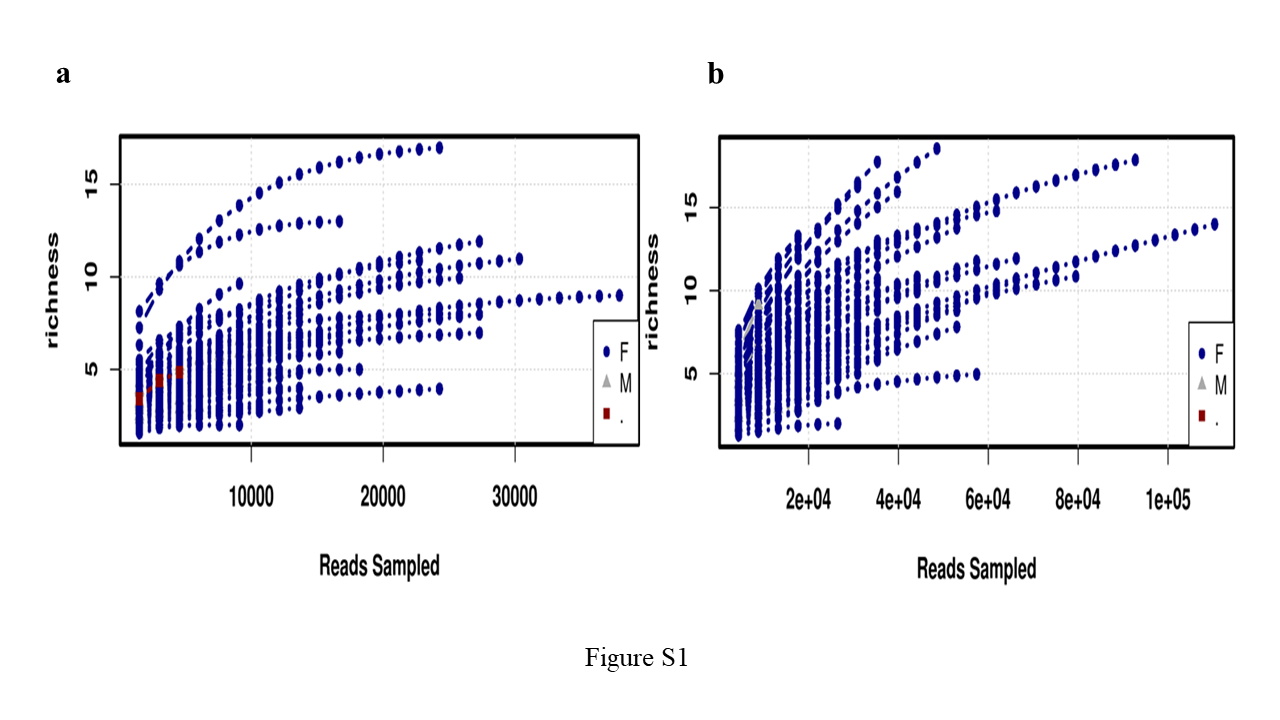

Supplement: Supplementary file 2 — Additional file 2: Figure S1. Rarefaction curve for all sequenced samples. a Data derived from sequencing of the V3V4 region. b Data derived from sequencing of the V4 region. The red squares represent the controls that were introduced during the experiments to ensure the success of the sequencing. Abbreviations: F, female; M, male. [file 13071_2019_3402_MOESM2_ESM.tif]

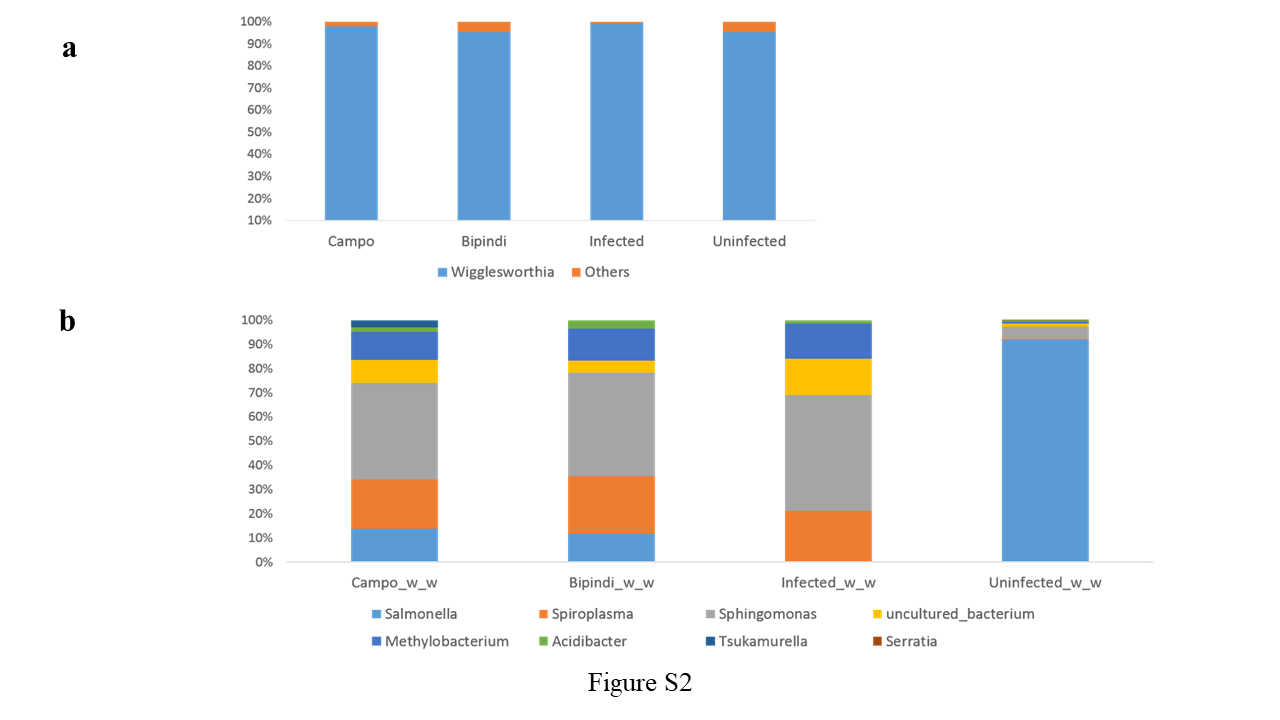

Supplement: Supplementary file 3 — Additional file 3: Figure S2. Summary of bacterial abundances in different conditions. a Abundances with Wigglesworthia. b Abundances without Wigglesworthia. Infected: positive with Trypanosoma congolense (s.l.). Abbreviation: w_w, without Wigglesworthia. [file 13071_2019_3402_MOESM3_ESM.tif]

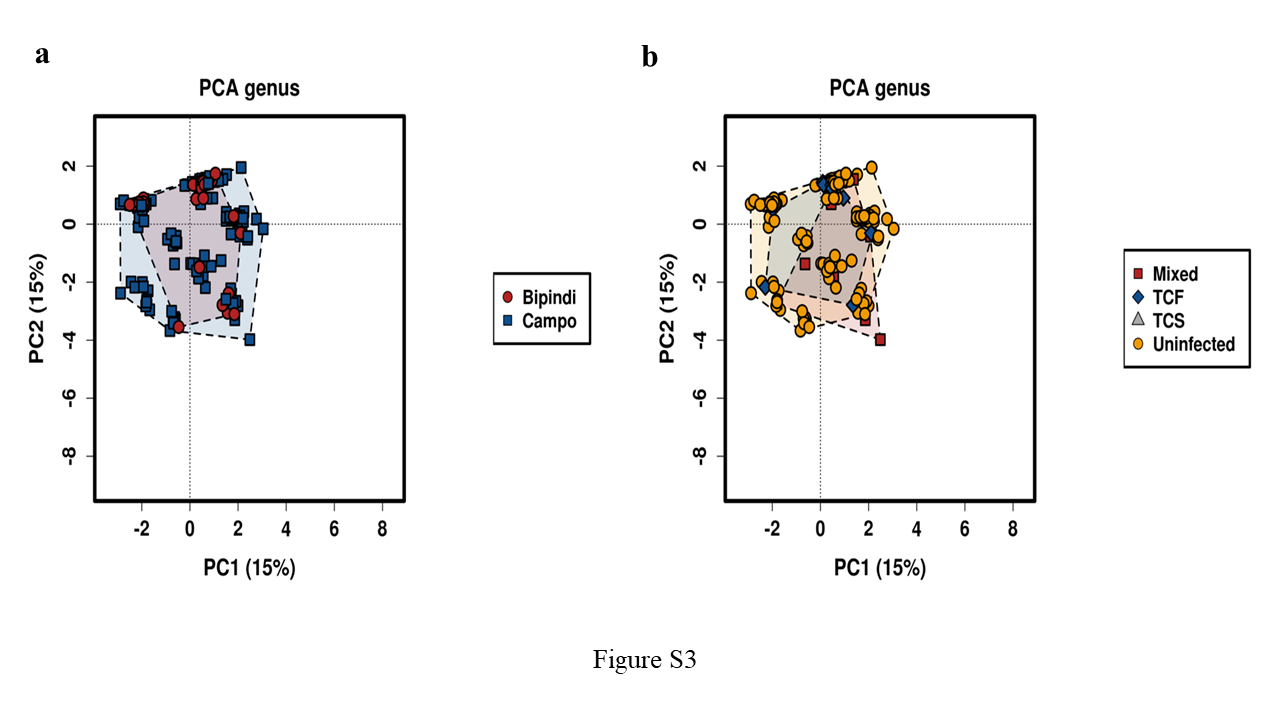

Supplement: Supplementary file 4 — Additional file 4: Figure S3. Principal components analysis (PCA) using the Bray-Curtis diversity index based on the focus origin (Campo or Bipindi) (a) or infection status (b). Key: Bipindi, flies harvested in Bipindi; Campo, flies harvested in Campo; TcF, T. congolense “forest” type; TcS, T. congolense “savannah” type; mixed infection, flies simultaneously positive by both parasites (TcF + TcS). [file 13071_2019_3402_MOESM4_ESM.tif]

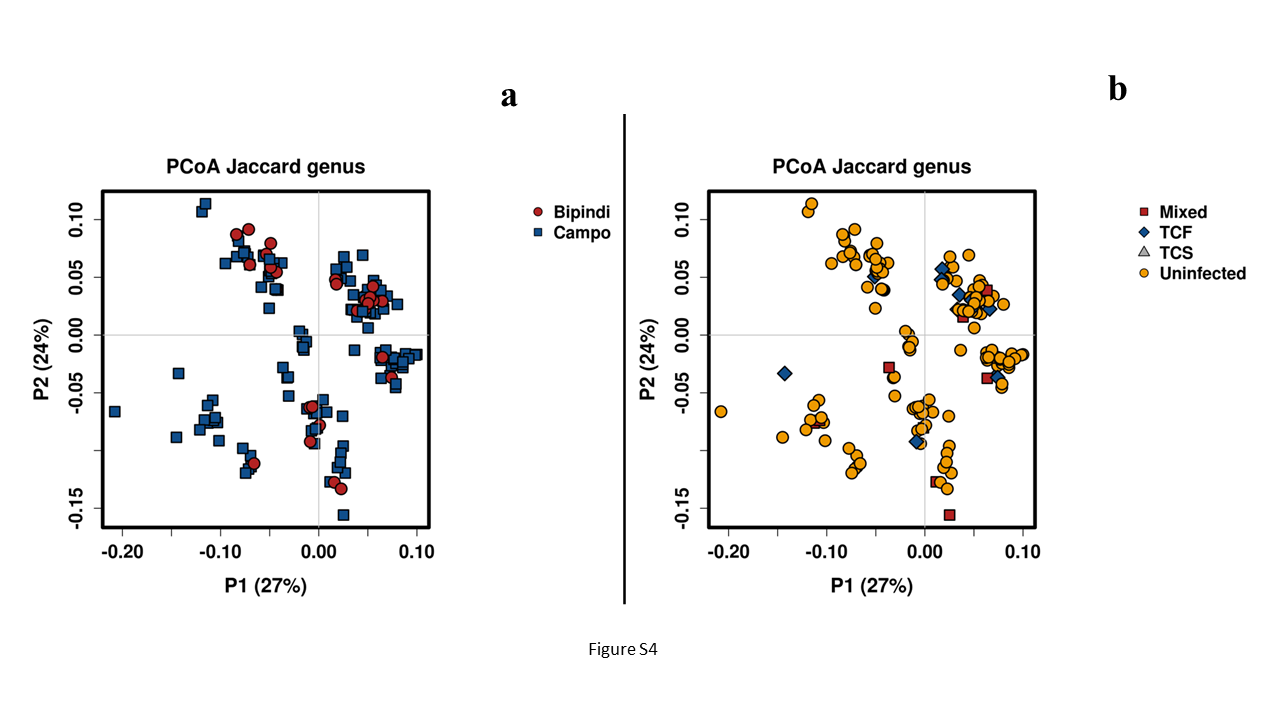

Supplement: Supplementary file 6 — Additional file 6: Figure S4. Principal components analysis (PCA) using the Jaccard diversity index based on the focus origin (Campo or Bipindi) (a) or infection status (b). Key: Bipindi, flies harvested in Bipindi; Campo, flies harvested in Campo; TcF, T. congolense “forest” type; TcS, T. congolense “savannah” type; mixed infection, flies simultaneously positive for both parasites (TcF + TcS). [file 13071_2019_3402_MOESM6_ESM.tif]

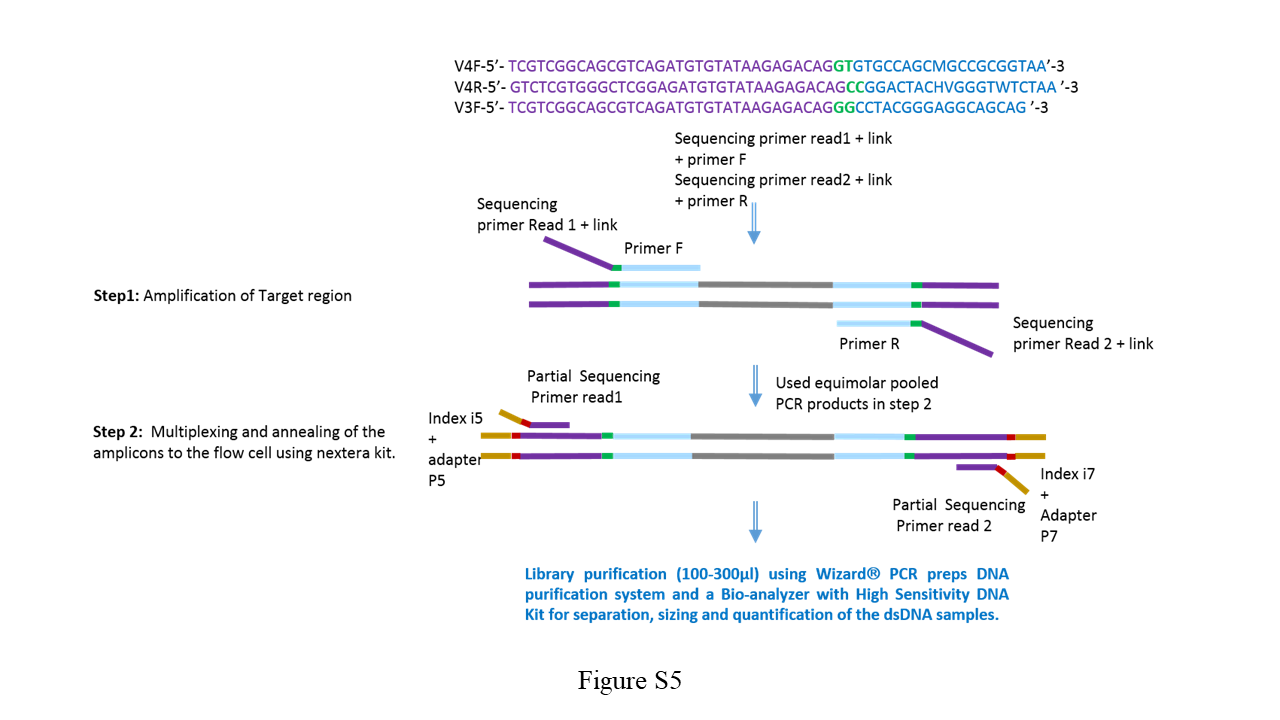

Supplement: Supplementary file 8 — Additional file 8: Figure S5. Workflow of the amplicon library construction. The target region of interest from the Nextera kit is shown. [file 13071_2019_3402_MOESM8_ESM.tif]
