# Supplementary material for: The composition and abundance of bacterial communities residing in the gut of Glossina palpalis palpalis captured in two sites of southern Cameroon
Source: Parasit Vectors. 2019 Apr 2;12:151. doi: 10.1186/s13071-019-3402-2 (PMC6444424; doi:10.1186/s13071-019-3402-2)
Supplement: Supplementary file 5 — Additional file 5: Table S2. Non-normalized abundance table (number of reads per sample). [file 13071_2019_3402_MOESM5_ESM.pdf]

Table S2a: non-normalized abundance table (number of reads (V4 region) per samples)

Samples	Wigglesworthia	Other bacteria
J1-10_S76	809	36
J1-11_S112	16300	6
J1-21_S88	16669	21
J1-23_S94	22786	79
J1-28_S14	27999	35
J1-31_S49	39896	133
J100-7_S38	59274	8
J11-6_S63	21381	10
J11-7_S71	7	6
J12-1_S56	17482	55
J15-4_S58	54733	72
J15-5_S119	415	13
J16-13_S103	22445	4
J16-14_S55	19417	23
J16-16_S39	1816	19
J16-1_S168	243	25
J16-23_S69	31895	17
J16-33_S158	23704	1
J16-34_S160	6763	2
J16-35_S75	20073	12
J16-37_S189	21890	11
J16-40_S64	94747	69
J16-41_S102	9526	16
J16-43_S21	5727	20
J16-45_S82	16203	52
J16-51_S50	65869	219
J16-53_S169	10706	4
J17-11_S24	3067	7
J17-20_S120	21031	9
J17-23_S81	20343	44
J17-27_S142	34042	21
J17-42_S12	14571	33
J17-6_S20	31353	20
J18-10_S40	1997	71
J18-11_S42	2940	21
J18-13_S148	21028	17
J18-14_S99	14300	10
J18-15_S166	45561	32
J18-16_S35	3833	259
J18-17_S72	17752	19
J18-18_S77	10382	6
J18-18bis_S17	7375	5
J18-24_S111	18218	2041
J18-25_S184	16520	3
J18-27_S122	17528	2
J18-34_S26	21880	15
J18-37_S53	69436	9

J18-38_S147	22966	9
J18-45_S175	13474	11
J18-9_S136	9957	4
J19-10_S87	10126	80
J19-17_S127	15382	20
J19-1_S157	13215	1
J19-20_S9	36150	36
J19-22_S182	9445	14
J2-5_S18	39434	10
J2-6_S186	4553	12
J2-7_S22	22111	8
J2-8_S151	11085	8
J20-10_S66	34678	11
J20-25_S30	31035	134
J20-27_S98	1430	0
J20-31_S29	29015	1
J20-34_S59	26968	7
J20-4_S95	164	45
J20-4bis_S101	149	2
J20-7_S45	20844	31
J20-9_S44	38676	18
J21-10_S37	56879	11
J22-13_S130	4513	12
J22-1_S183	6229	5
J22-2_S152	3338	14
J22-7_S143	21113	20
J23-6_S92	9333	16
J23-7_S114	20876	2
J24-10_S36	19544	40
J25-3_S164	48043	45
J26-1_S17	18153	13
J26-2_S128	13996	3
J27-13_S190	31006	5
J28-10_S150	2333	7
J28-5_S149	7294	15
J28-9_S91	12691	3
J29-16_S80	8488	25
J29-17_S61	9938	39
J29-17bis_S13	8318	3
J3-4_S181	16377	70
J30-19_S170	54958	2
J30-6_S165	9993	12
J30-8_S133	3058	33
J30-9_S83	14382	10
J31-15_S16	38516	10
J31-15bis_S10	17529	10
J31-18_S1	46586	725
J31-25_S6	38884	14
J31-26_S138	26292	10
J32-2_S25	23436	74

J32-5_S153	29938	13
J33-13_S100	13548	7
J34-16_S106	26473	3
J34-2_S47	17896	9
J34-2bis_S118	25049	17
J34-3_S33	67066	21
J34-4_S46	9661	39
J35-15_S15	30992	12
J35-8_S129	16476	14
J35-9_S74	18957	16
J36-13_S154	18349	5
J36-1_S105	25023	6
J36-3_S173	11934	2
J37-1_S115	38915	19
J37-1bis_S62	110312	69
J38-1_S134	5102	25
J38-3_S51	59617	23
J39-4_S161	24287	1
J39-6_S68	12390	46
J4-10_S176	35515	36
J4-16_S31	16761	24
J4-17_S32	32127	26
J4-25_S146	7702	7
J4-32_S121	551	7
J40-10_S178	4052	2
J40-11_S135	4385	29
J40-14_S28	25964	18
J40-1a_S34	267	12
J40-1b_S97	6394	8
J40-2_S79	6501	40
J40-4_S163	11296	4
J40-7_S162	47002	13
J41-5_S93	15769	60
J41-8_S167	15236	5
J42-4_S132	5619	1
J42-6_S113	9938	10
J45-3_S117	19055	7
J46-3_S23	35944	2
J47-12_S84	27149	6
J47-16_S13	41415	2
J47-17_S43	19339	458
J47-20_S174	50964	100
J47-23_S159	13260	17
J47-2_S116	16966	16
J47-2bis_S48	29735	40
J47-31_S137	43043	4
J47-6_S4	41395	7
J47-9_S78	27558	11
J47-9bis_S180	24602	2
J48-4_S3	43774	11

J52-14_S171	19161	3
J52-6_S124	20423	0
J55-14_S5	38259	50
J55-43_S86	20676	13
J56-1_S177	3078	8
J58-1_S187	2411	5
J58-24_S140	28287	14
J58-2_S185	33748	147
J59-4_S73	24584	10
J6-2_S144	25273	62
J6-3_S27	961	13
J6-5_S145	30975	1
J60-18_S172	45988	223
J65-36_S110	30577	50
J68-3_S139	2948	9
J68-9_S126	21622	25
J7-1_S57	6002	53
J7-1bis_S191	9880	3
J7-9_S89	23723	25
J70-8_S107	10808	18
J73-49_S125	25895	0
J73-51_S123	16717	7
J73-68_S7	22761	161
J77-12_S8	16824	22
J78-56_S70	27363	16
J79-43_S156	8409	5
J79-98_S65	39059	176
J79-99_S67	36661	15
J8-5_S10	23726	54
J8-6_S11	11618	15
J8-9_S60	21642	77
J80-115_S52	82340	21
J80-116_S155	25209	3
J80-117_S54	27970	1
J83-3_S108	22823	9
J84-21_S85	28453	0
J88-1_S109	18258	120
J89-8_S141	32	5
J9-6_S41	28293	26
J9-8_S90	20264	33
J9-9_S2	18670	1
J94-4_S188	35329	1
J95-11_S19	22990	22
NTC-1_S96	10	19
NTC-2_S192	281	8
total	4227129	8241

Table S2b: non-normalized abundance table (number of reads (V3V4 region) per samples)

Samples	Wigglesworthia	Others bacteria
J1-10_S76	183	10
J1-11_S112	8995	2
J1-21_S88	3996	10
J1-23_S94	8330	22
J1-28_S14	13561	7
J1-31_S49	13105	62
J100-7_S38	17800	4
J11-6_S63	3409	4
J11-7_S71	7	12
J12-1_S56	4219	45
J15-4_S58	12386	13
J15-5_S119	158	14
J16-13_S103	14473	2
J16-14_S55	4126	5
J16-16_S39	465	6
J16-1_S168	172	3
J16-23_S69	11052	12
J16-33_S158	12266	2
J16-34_S160	3581	3
J16-35_S75	4718	4
J16-37_S189	3825	2
J16-40_S64	24113	24
J16-41_S102	6035	8
J16-43_S21	1450	11
J16-45_S82	2369	6
J16-51_S50	15286	41
J16-53_S169	9077	3
J17-11_S24	892	5
J17-20_S120	11004	0
J17-23_S81	4191	4
J17-27_S142	17272	309
J17-42_S12	4070	8
J17-6_S20	7299	6
J18-10_S40	570	3
J18-11_S42	490	16
J18-13_S148	9994	25
J18-14_S99	9927	0
J18-15_S166	27647	32
J18-16_S35	1319	72
J18-17_S72	5129	6
J18-18_S77	2972	3
J18-18bis_S17	10565	5
J18-24_S111	6978	10
J18-25_S184	9191	3
J18-27_S122	11978	3
J18-34_S26	3606	8
J18-37_S53	13057	2

J18-38_S147	13784	11
J18-45_S175	13462	16
J18-9_S136	5448	3
J19-10_S87	4063	6
J19-17_S127	6469	10
J19-1_S157	6099	1
J19-20_S9	8629	1
J19-22_S182	5073	4
J2-5_S18	14455	5
J2-6_S186	2583	3
J2-7_S22	11107	7
J2-8_S151	9079	2
J20-10_S66	5828	2
J20-25_S30	7975	25
J20-27_S98	9591	4
J20-31_S29	11228	0
J20-34_S59	3360	2
J20-4_S95	62	29
J20-4bis_S101	56	11
J20-7_S45	3995	5
J20-9_S44	8885	7
J21-10_S37	16042	4
J22-13_S130	3787	0
J22-1_S183	3567	2
J22-2_S152	2217	1
J22-7_S143	12884	8
J23-6_S92	2990	2
J23-7_S114	13521	2
J24-10_S36	3237	7
J25-3_S164	26607	38
J26-1_S17	1639	10
J26-2_S128	11218	0
J27-13_S190	14335	1
J28-10_S150	1412	1
J28-5_S149	4231	7
J28-9_S91	4405	0
J29-16_S80	5878	22
J29-17_S61	1271	8
J29-17bis_S13	3610	2
J3-4_S181	12022	54
J30-19_S170	37903	2
J30-6_S165	7487	4
J30-8_S133	1582	31
J30-9_S83	3605	5
J31-15_S16	7298	3
J31-15bis_S10	7733	0
J31-18_S1	1438	14
J31-25_S6	9759	1
J31-26_S138	15451	0
J32-2_S25	7555	19

J32-5_S153	24619	245
J33-13_S100	15219	2
J34-16_S106	13770	2
J34-2_S47	3711	4
J34-2bis_S118	10732	5
J34-3_S33	11117	6
J34-4_S46	2819	8
J35-15_S15	6890	8
J35-8_S129	11315	0
J35-9_S74	5614	4
J36-13_S154	9568	3
J36-1_S105	14765	1
J36-3_S173	10010	3
J37-1_S115	8532	7
J37-1bis_S62	30765	27
J38-1_S134	3850	17
J38-3_S51	5553	0
J39-4_S161	18998	0
J39-6_S68	3142	3
J4-10_S176	15053	14
J4-16_S31	3920	3
J4-17_S32	8686	18
J4-25_S146	4473	2
J4-32_S121	155	8
J40-10_S178	5007	4
J40-11_S135	2351	6
J40-14_S28	7644	16
J40-1a_S34	53	7
J40-1b_S97	10932	9
J40-2_S79	1455	12
J40-4_S163	10591	1
J40-7_S162	27215	5
J41-5_S93	5435	436
J41-8_S167	13065	4
J42-4_S132	6863	2
J42-6_S113	2678	2
J45-3_S117	8814	5
J46-3_S23	13170	1
J47-12_S84	9019	3
J47-16_S13	9975	0
J47-17_S43	3642	82
J47-20_S174	11954	14
J47-23_S159	7541	6
J47-2_S116	7224	3
J47-2bis_S48	7563	9
J47-31_S137	28124	0
J47-6_S4	7526	3
J47-9_S78	7883	1
J47-9bis_S180	14838	4
J48-4_S3	12025	6

J52-14_S171	10984	0
J52-6_S124	13001	3
J55-14_S5	8588	4
J55-43_S86	7050	10
J56-1_S177	2480	1
J58-1_S187	1296	0
J58-24_S140	8628	2
J58-2_S185	833	16487
J59-4_S73	9146	1
J6-2_S144	14507	29
J6-3_S27	316	11
J6-5_S145	17844	0
J60-18_S172	27903	141
J65-36_S110	10813	17
J68-3_S139	2320	9
J68-9_S126	13343	13
J7-1_S57	1066	17
J7-1bis_S191	6185	2
J7-9_S89	9460	13
J70-8_S107	3065	5
J73-49_S125	9328	1
J73-51_S123	6298	1
J73-68_S7	7335	69
J77-12_S8	5316	5
J78-56_S70	6673	6
J79-43_S156	5779	1
J79-98_S65	2603	11
J79-99_S67	9974	9
J8-5_S10	6030	30
J8-6_S11	3487	8
J8-9_S60	6043	9
J80-115_S52	25498	3
J80-116_S155	12874	1
J80-117_S54	9840	0
J83-3_S108	9812	0
J84-21_S85	11764	3
J88-1_S109	8324	2
J89-8_S141	6	0
J9-6_S41	9545	21
J9-8_S90	3984	5
J9-9_S2	6933	9
J94-4_S188	1385	0
J95-11_S19	2144	25
Total	1578889	19274
%tage	98,79565944	1,20600965
