# Supplementary material for: The composition and abundance of bacterial communities residing in the gut of Glossina palpalis palpalis captured in two sites of southern Cameroon
Source: Parasit Vectors. 2019 Apr 2;12:151. doi: 10.1186/s13071-019-3402-2 (PMC6444424; doi:10.1186/s13071-019-3402-2)
Supplement: Supplementary file 7 — Additional file 7: Table S3. Primers used for PCR amplification of trypanosomes. [file 13071_2019_3402_MOESM7_ESM.pdf]

Table S3. Primers used for PCR amplification of trypanosomes.

Species	Primer sequence	Amplified product length	Reference
T. brucei s.l.	5'-CGAATGAATATTAAACAATGCGCAG-3'<br>5'-AGAACCATTTATTAGCTTTGTTGC-3'	164 bp	Masiga et al. [70]
T. congolense ("forest" type)	5'-CGAATGAATATTAAACAATGCGCAG-3'<br>5'-AGAACCATTTATTAGCTTTGTTGC-3'	350 bp	Masiga et al. [70]
T. congolense ("savannah" type)	5'-CGAATGAATATTAAACAATGCGCAG-3'<br>5'-AGAACCATTTATTAGCTTTGTTGC-3'	341 bp	Moser et al. [71]

bp : base pairs.
